# A Strep A vaccine global demand and return on investment forecast to inform industry research and development prioritization

**DOI:** 10.1038/s41541-023-00690-2

**Published:** 2023-08-09

**Authors:** Donald R. Walkinshaw, Meghan E. E. Wright, Marni Williams, Tanya M. F. Scarapicchia, Jean-Louis Excler, Ryan E. Wiley, Anne E. Mullin

**Affiliations:** 1 Shift Health, Toronto, Canada; 2 International Vaccine Institute, Seoul, Republic of Korea

**Keywords:** Bacterial infection, Market analysis

## Abstract

Investment in Strep A vaccine R&D is disproportionately low relative to the large burden of Strep A diseases globally. This study presents a novel Strep A vaccine global demand and financial forecast model with estimates of potential global demand and associated revenue and profits for a hypothetical Strep A vaccine as well as a net present value (NPV) analysis of return on capital investments required to develop the vaccine. A positive NPV was calculated for a variety of developer scenarios and target populations, including the global rollout of the vaccine in private and public markets by a multinational pharmaceutical corporation and a staged rollout by a developing country vaccine manufacturer for both infant and child populations. The results suggest there is a viable commercial market for a Strep A vaccine. It is hoped that this study will help to inform industry decision-making and drive increased prioritization of, and investment in, Strep A vaccine research and development.

## Introduction

*Streptococcus pyogenes* (Group A Streptococcus or “Strep A”) is a gram-positive bacterium that causes a wide spectrum of diseases in humans. The most common Strep A disease is pharyngitis, which results in over 600 million new global cases per year, predominantly in children^1^. Strep A infection can also lead to serious invasive (e.g., sepsis) and toxin-mediated (e.g., scarlet fever) diseases, as well as necrotizing fasciitis, toxic shock syndrome, poststreptococcal glomerulonephritis, acute rheumatic fever (ARF) and rheumatic heart disease (RHD). Of Strep A diseases, RHD has the greatest morbidity and mortality, with global disability-adjusted life years (DALYs) of 10.5 million, mostly from premature deaths, and a global age-standardized prevalence rate of 513.7 per 100 000 people^2^. In a recent study, RHD-related deaths were forecasted to remain almost constant until 2040, unlike other high-burden communicable diseases, including HIV and tuberculosis, for which mortality is projected to decrease quite significantly by 2040^3^. It is estimated that Strep A causes 639 000 deaths each year, with RHD-related deaths accounting for approximately 73% of those deaths and invasive disease due to Strep A infection accounting for 25%^4^. People in low- and middle-income countries as well as high-risk populations including pregnant women, the elderly, and Indigenous populations are disproportionately affected by diseases associated with Strep A infection. Strep A disease management involves antibiotic treatment and there is currently no pharmacological option to prevent Strep A infections.

Despite clear evidence that a vaccine against Strep A is possible—including evidence of acquired natural immunity to Strep A infections, extensive preclinical data in animal models, and proof-of-principle studies with human challenge models—to date, only three vaccine candidates currently in development have completed or recently begun a Phase 1 clinical trial^5,6^. The clinical development of Strep A vaccines has been impeded by many challenges, including: unanswered scientific questions (e.g., absence of putative vaccine-induced correlate(s) of protection, the impact of high strain diversity on the breadth of vaccine efficacy); gaps in product development technologies (e.g., absence of high-throughput, standardized assays to measure Strep A vaccine-induced immune responses); and an unclear regulatory pathway (e.g., what efficacy and immunogenicity endpoints, target populations and study designs will be expected by regulators). In addition, safety concerns stemming from serious adverse events during Strep A vaccine candidate testing in the late 1960s led to an approximately 30-year US FDA-imposed ban on Strep A vaccine testing in humans, setting the field back by decades^7,8^. The field has also suffered from a lack of private and public market prioritization and access to funding—exemplified by the substantial underfunding of Strep A/RHD R&D funding relative to its disease burden^9^.

To begin addressing these issues, the WHO led the development of Preferred Product Characteristics (PPCs) for a Strep A vaccine and an R&D Roadmap for Strep A vaccine development to promote alignment across the field around Strep A vaccine profile considerations as well as clinical development activities and enablers^7^. Building on these important steps, and with funding from the Wellcome Trust, the Strep A Vaccine Global Consortium (SAVAC; https://savac.ivi.int/) was established by the International Vaccine Institute and stakeholders from the Murdoch Children’s Research Institute (Australia), Telethon Kids Institute (Australia), Harvard T.H. Chan School of Public Health (United States), PATH (United States), Imperial College London (United Kingdom), University of Cape Town (Republic of South Africa), University of Colorado (United States) and Shift Health (Canada). SAVAC’s mission is to increase awareness of the need for a Strep A vaccine and promote greater engagement and investment from diverse stakeholders in Strep A vaccine development. A major early priority for SAVAC has been the development of a Full Value of Vaccines Assessment (FVVA) to quantify and articulate the value of a Strep A vaccine from multiple angles including the health and economic burden of Strep A disease, the public health impact of a hypothetical Strep A vaccine as well as the business case for investment in Strep A vaccine development^10^.

Quantifying the potential demand and market for a Strep A vaccine is important to inform investment decisions by vaccine developers and manufacturers—whether pharmaceutical multinational corporations (Pharma MNC) or developing country vaccine manufacturers (DCVMs)—particularly as Strep A vaccine development has yet to garner significant attention from biopharmaceutical companies^6^. This study advances a novel Strep A vaccine demand and financial forecast model that provides an estimate for the potential demand and associated revenue and profit for a hypothetical Strep A vaccine globally as well as a net present value (NPV) analysis of return on investments required for the development, licensure, and manufacturing of a Strep A vaccine. While biopharmaceutical companies have unique and proprietary forecasting methods and criteria for investment decisions, it is hoped that the results of this study—the first vaccine demand and return on investment analysis published for a Strep A vaccine—will help to inform industry decision-making and drive increased prioritization of Strep A vaccine development as a viable commercial opportunity.

## Results

The following Strep A vaccine demand and financial forecast model leverages traditional demand and return on investment methodological approaches^11–19^, a landscape assessment of Strep A vaccines in development^6^, information from proxy vaccines^20,21^, as well interviews with infectious disease and vaccine experts, global health funders, in-country vaccine decision-makers and representatives from Pharma MNCs and DCVMs. Information gathering was used to inform key assumptions, highlighted here, and described in detail in the Methods section. Results presented herein include annual demand (total number of doses), revenue, profit, and a return on capital investment analysis for a variety of scenarios, including public (through infant or child immunization programs) and private market delivery as well as global rollout by a Pharma MNC and staged rollout by a DCVM.

### Timing of introduction analysis

Based on typical R&D timelines^22,23^ and validated through expert interviews, the year of first market entry for the hypothetical Strep A vaccine is assumed to be 2033 (and for Gavi-eligible countries, 2035; see below). Starting in that year, the vaccine is assumed to be available through the private market in all countries (with level and rate of uptake via the private market driven by country income level). Also beginning in 2033, early-adopting countries are assumed to begin including the vaccine into their national immunization programs (NIP), with other countries following a timeline based on a scoring system driven by a combination of local Strep A disease burden, track record of new vaccine adoption and overall vaccine delivery infrastructure (Supplementary Table 1). For each of those dimensions, country-specific data was used to assign a relative “score” per country, which was then translated directly into a number of years until introduction in that country post first regulatory approval, based on an expected maximum of 15 years for complete global introduction of the vaccine. In this way, the slowest adopting country would begin introducing the vaccine through its NIP by 2047. RHD was used as an indicator of Strep A disease burden due the associated high morbidity and mortality relative to other Strep A diseases, an important public health consideration for in-country decision-makers. In addition, RHD is the only Strep A disease that currently has detailed country-level epidemiological data readily available.

In some cases, the year of introduction was then manually adjusted based on: assumed developer priorities (i.e., reflecting the degree to which the Pharma MNC or DCVM would prioritize launching in certain countries based on commercial capacity and vaccine market dynamics); signals of countries prioritizing Strep A disease management and/or Strep A vaccine R&D (e.g., Australia’s investment in The Australian Strep A Vaccine Initiative [ASAVI] which is supporting efforts in vaccine development, disease surveillance and community engagement)^24^; as well as time required for Gavi-eligible countries to qualify for and receive access to the vaccine through the Gavi program. It is assumed that Gavi would support the introduction of the Strep A vaccine in Gavi-eligible countries, of which there are 31 low-income countries (LICs) and 18 lower middle-income countries (LMICs; as of 2021), and that the process of qualifying for Gavi and UNICEF procurement commitment would require an additional 2 years beyond first licensure. Thus, the first year of market entry for Gavi countries was assumed to be 2035 rather than 2033, and the specific year of introduction for Gavi countries was determined based on the analysis described above for other countries. Two different vaccine developer scenarios are presented herein: (1) Global rollout by a Pharma MNC starting in 2033, and (2) Staged rollout by a DCVM starting in 2033 (all countries except high-income countries [HICs]) and 2038 (HIC markets). A third scenario in which Pharma MNCs similarly engage in a staged rollout that delays launch in HICs was also explored. In this case, which accounts for the possibility that the perceived need for a Strep A vaccine in HICs is not sufficiently recognized initially, it is assumed that Pharma MNCs may engage in a staged rollout wherein the vaccine is introduced in lower-income countries (because they account for a greater proportion of Strep A burden) before market launch in HICs beginning 5 years later. Although not modeled in this study, another scenario with potential advantages would be a hybrid model in which a Pharma MNC partners with a DCVM to leverage the lower R&D costs of DCVMs while benefiting from the global commercialization reach of a Pharma MNC.

**Fig. 1:**
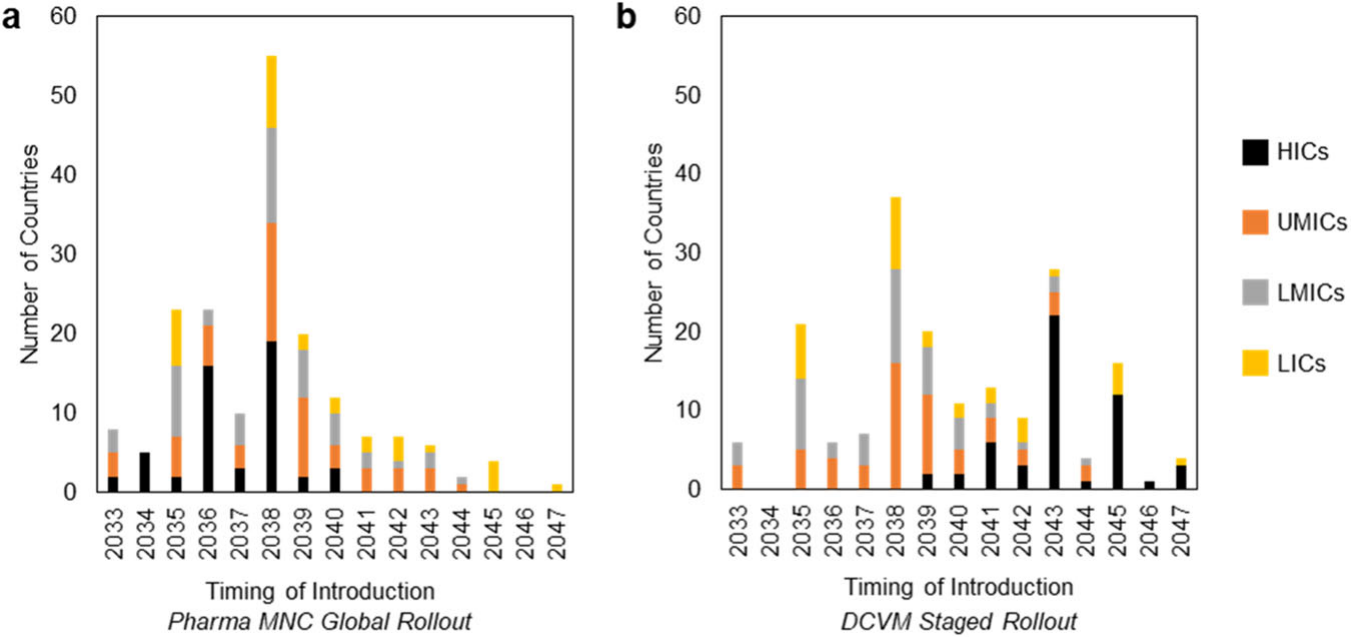
Number of countries introducing the vaccine in the public market each year segmented by country-income level. Overview of timing of introduction in the public market (national immunization program; NIP), segmented by country income-level group for **a** the Pharma MNC Global Rollout scenario and **b** the DCVM Staged Rollout scenario. HICs high-income countries, UMICs upper middle-income countries, LMICs lower middle-income countries, LICs low-income countries.

Results from the model indicate that countries will collectively introduce the vaccine over a period of 15 years for all Pharma MNC and DCVM scenarios. In the Pharma MNC Global Rollout scenario, approximately 70% of countries introduce the vaccine by 2038 and about 90% introduce the vaccine by 2041, compared to just over 40% of countries by 2038 and 65% of countries by 2041 in the DCVM Staged Rollout scenario. Figure 1 shows the distribution of year of adoption into each country’s NIP, grouped by country income-level group for the Pharma MNC Global Rollout and DCVM Staged Rollout scenarios (panel a and b, respectively). Country income-level groups include LICs, LMICs, upper middle-income countries (UMICs), and HICs. Details on the data inputs and results of the timing of introduction analysis for each country included in the analysis are reported in the Supplementary Tables and Figures. Early adopters include LICs and LMICs with high RHD burden and strong history of new vaccine adoption and vaccine delivery infrastructure. In the Pharma MNC Global Rollout scenario, the vaccine is expected to be adopted early by major pharmaceutical markets including the US and 5EU (i.e., United Kingdom, France, Germany, Italy, Spain) based on historical precedence of Pharma MNCs targeting these high-priority commercial markets for initial launches. Though RHD burden is relatively low in these markets, other Strep A diseases such as pharyngitis, impetigo, and Scarlet fever drive health, economic, and broader societal burden in HICs^1,25–27^ Late adopters in the Pharma MNC Global Rollout scenario are mostly UMICs and LMICs with moderate RHD burden, poor new vaccine adoption history, and moderate vaccine delivery infrastructure scores. In the Pharma MNC Staged Rollout Scenario, HICs adopt in the same sequence but starting in 2038. In the DCVM Staged Rollout scenario, it is assumed that the DCVM initially targets lower-income countries, followed by non-US and non-5EU HICs, and then later the major US and 5EU pharmaceutical markets. The timing of the introduction of specific countries within these groups is based on new vaccine adoption history and vaccine delivery infrastructure scores as well as evidence of Strep A prioritization as a country-level public health priority.

Two additional scenarios were explored for the Pharma MNC Global Rollout scenario: an Optimistic Timeline scenario in which it is assumed that development timelines, as well as NIP introduction, may be accelerated (in part due to the precedent set by COVID-19 vaccine development and rollout and in part due to the assumption that countries will gradually improve vaccine delivery infrastructure over the coming decade); and a Conservative Timeline scenario, in which the maximum number of years over which countries introduce the vaccine into their NIPs aligns with historical examples of vaccines that took longer for widespread adoption, such as HepB or HiB^20^. Under the Optimistic Timeline for the Pharma MNC Global Rollout scenario, market entry is in the year 2030 and 100% of countries have introduced the vaccine in their NIP by 2038. Under the Conservative Timeline, market entry is in the year 2033 and less than half of countries have introduced the vaccine in their NIP by 2038 (100% of countries by 2050).

### Annual demand

Within each developer scenario (Pharma MNC and DCVM), two different target populations are explored: Infants (<1 year) and young children (between 4 and 7 years) because it is not yet known which of these two target populations will ultimately be prioritized for future Strep A vaccination. Annual Strep A vaccine demand by the number of doses (assuming a 3-dose primary regimen and no boosters) was estimated using country-level proxy vaccine coverage (third dose of diphtheria, tetanus, and pertussis vaccine (DTP3) for the infant population and second dose of each country’s measles-containing vaccine (MCV2) for the young children population) and a wastage rate of 5%^28,29^.

**Fig. 2:**
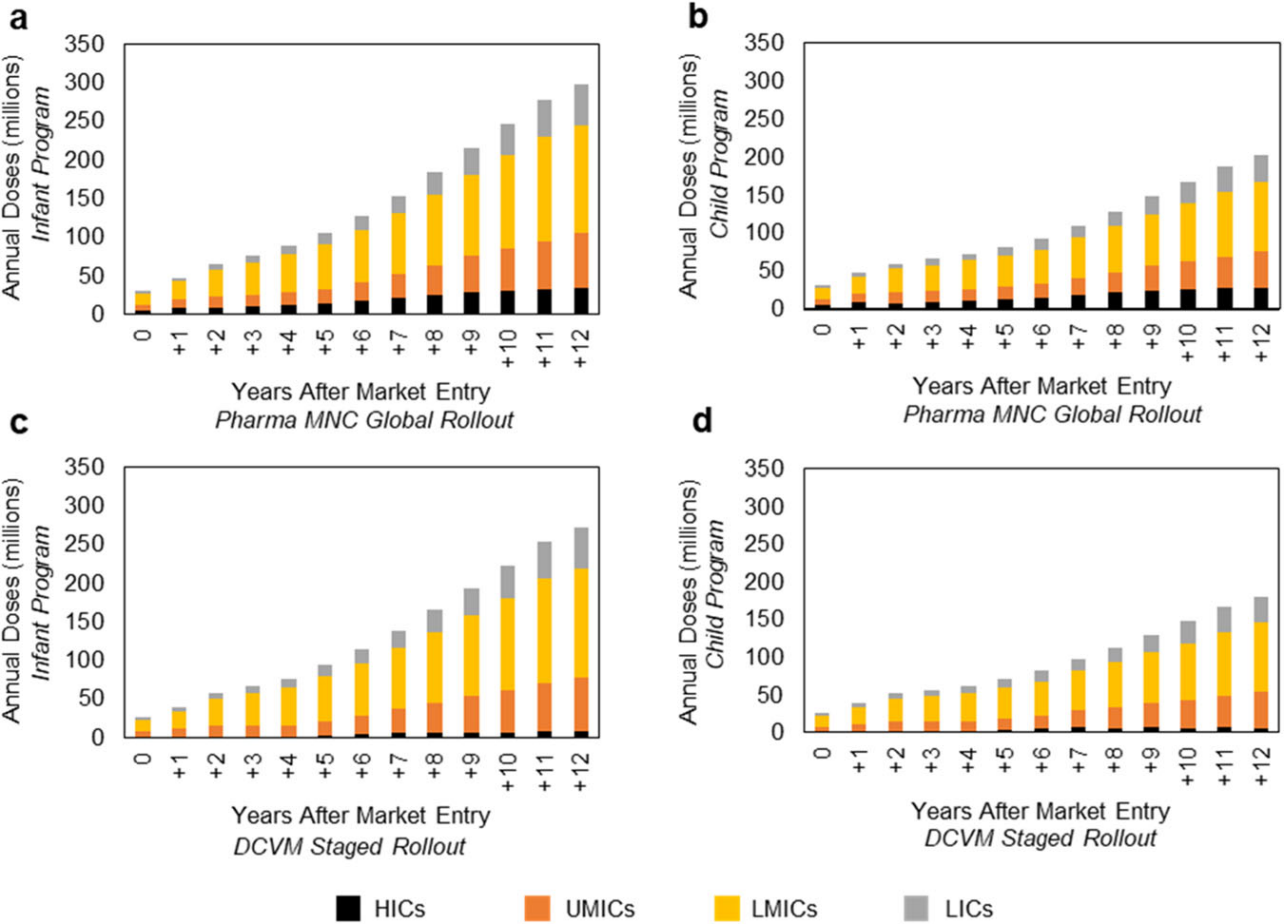
Annual number of doses forecasted. Annual doses in millions delivered throughout the forecast period segmented by country income-level group for **a** the Pharma MNC Global Rollout infant immunization program scenario, **b** the Pharma MNC Global Rollout child immunization program scenario, **c** the DCVM Staged Rollout infant immunization program scenario, and **d** the DCVM Staged Rollout child immunization program. HICs high-income countries, UMICs upper middle-income countries, LMICs lower middle-income countries, LICs low-income countries.

Model results indicate that the total annual demand at year 12 for the Pharma MNC Global Rollout developer scenario is 298 million doses for the infant immunization program scenario and 202 million doses for child immunization (Fig. 2a, b, respectively). Annual demand at year 12 for the DCVM Staged Rollout scenario is 272 million doses for the infant immunization program scenario and 180 million for child immunization (Fig. 2c, d, respectively). (See Supplementary Tables 7, 8 for annual demand results at year 12 for additional scenarios). While immunizing infants would result in higher peak demand due to the relatively higher uptake of vaccines in the infant schedule compared to school-aged immunization programs, ensuring protection to peak years for pharyngitis incidence (~5–15 years) in the infant scenario would depend on greater vaccine durability than for the child immunization scenario (the hypothetical Strep A vaccine profile assumes at least 10 years of durability). For both the infant and child immunization programs, demand from LMICs contributes ~50% of the total annual doses at year 12 for both the Pharma MNC Global Rollout and DCVM Staged Rollout developer scenarios. The higher vaccine demand in LMICs is driven by population size (~2 × total population of UMICs) as well as the relatively higher Strep A burden (as measured by RHD incidence) resulting in earlier adoption in these countries compared to those with lower burden (HICs and UMICs).

### Annual revenue and profit

**Fig. 3:**
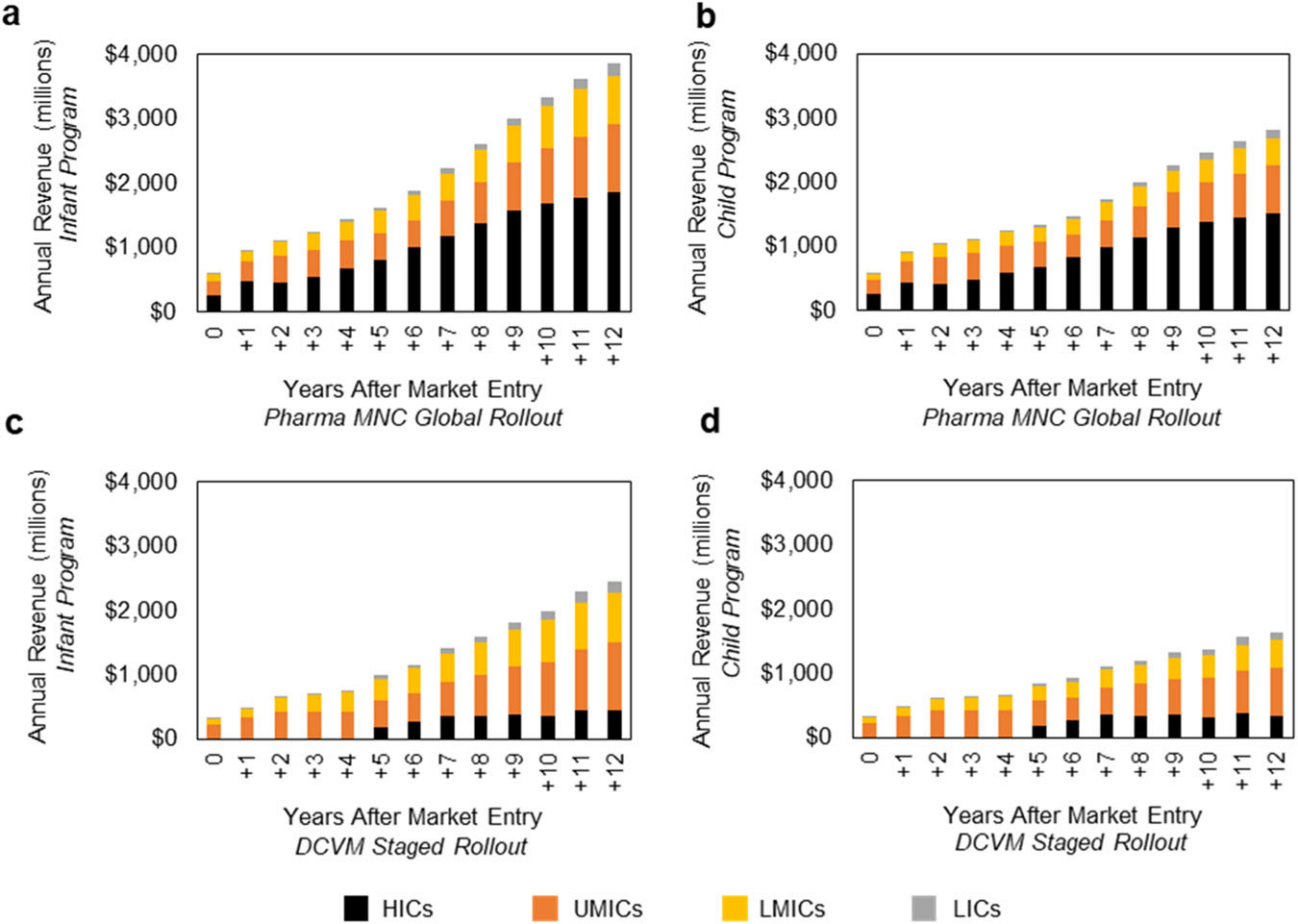
Annual revenue forecasted. Annual revenue (millions, USD) segmented by country income-level group for **a** the Pharma MNC Global Rollout infant immunization program scenario, **b** the Pharma MNC Global Rollout child immunization program scenario, **c** the DCVM Staged Rollout infant immunization program scenario, and **d** the DCVM Staged Rollout child immunization program. HICs high-income countries, UMICs upper middle-income countries, LMICs lower middle-income countries, LICs low-income countries.

**Fig. 4:**
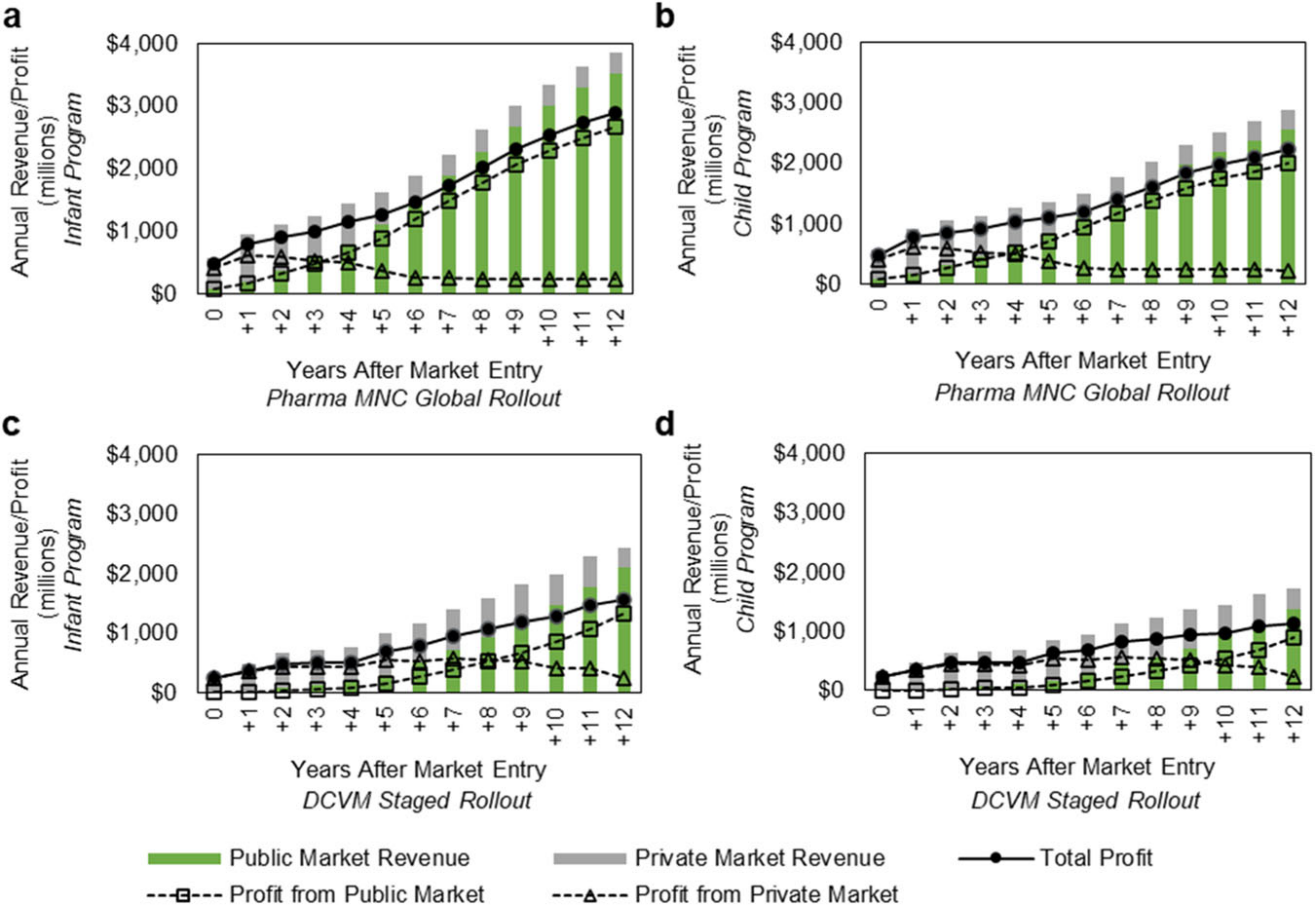
Annual revenue and profit forecasted by market type. Annual revenue and profit (millions, USD) segmented by private and public markets for **a** the Pharma MNC Global Rollout infant immunization program scenario, **b** the Pharma MNC Global Rollout child immunization program scenario, **c** the DCVM Staged Rollout infant immunization program scenario, and **d** the DCVM Staged Rollout child immunization program.

Annual revenue and profit were calculated based on assumed values of vaccine price and cost per dose, informed by historical price data for pneumococcal conjugate vaccine (PCV13) sourced from the Market Information for Access to Vaccines database (MI4A)^21^. Price and cost per dose are dependent on the vaccine presentation (i.e., vial or pre-filled syringe), which in turn is market dependent (e.g., public market vs. private market). As shown in Fig. 3, HIC markets contribute the greatest proportion of annual revenue at year 12 in the Pharma MNC Global Rollout developer scenario (approximately $1.9 billion USD or 48% and $1.5 billion or 53% for the infant and child immunization programs, respectively) due to the higher vaccine prices in these markets (e.g., $53.95 for pre-filled syringe in HICs vs. $3.40 for 1-dose vial in LICs). LIC markets represent only a small portion of annual revenue (less than 5% of total). In the DCVM Staged Rollout scenario, most of the revenue at year 12 is derived from UMICs (approximately $1 billion or 43% and $0.8 billion or 46% for the infant and child immunization programs, respectively) due to relatively higher vaccine prices in these markets. In the Pharma MNC Global Rollout scenario, about 90% of the annual profit at year 12 for both the infant and child immunization programs is derived from HIC and UMIC markets due to the higher price for the vaccine in these markets (Fig. 4a, b). For the DCVM scenario, HIC and UMIC market profits for both the infant and child immunization scenarios make up ~80% of the annual profit at year 12 due to lower uptake in HICs at this stage in the rollout (Fig. 4c, d).

Vaccine demand from both public markets and private markets was forecasted. It was assumed that the vaccine would be available in the private market immediately upon regulatory approval and would command a higher price, though demand would decrease in a given country once the vaccine became available via that country’s public NIP (See Methods for details on assumed coverage and ramp-up times for public and private market segments). Given forecasted slower ramp-up of vaccine coverage in the public market, the private market accounts for the majority of doses and the majority of revenue and profit until year 4 and 5 of the infant and child immunization programs, respectively, in the Pharma MNC Global Rollout developer scenario (Fig. 4a, b). Due to the slower rollout in HICs in the DCVM scenario, most of the revenue and profit is derived from the private market up until year 9 and 10 for the infant and child immunization scenarios, respectively (Fig. 4c, d). (See Supplementary Tables 7 and 8 for annual revenue and profit at year 12 in additional scenarios).

**Fig. 5:**
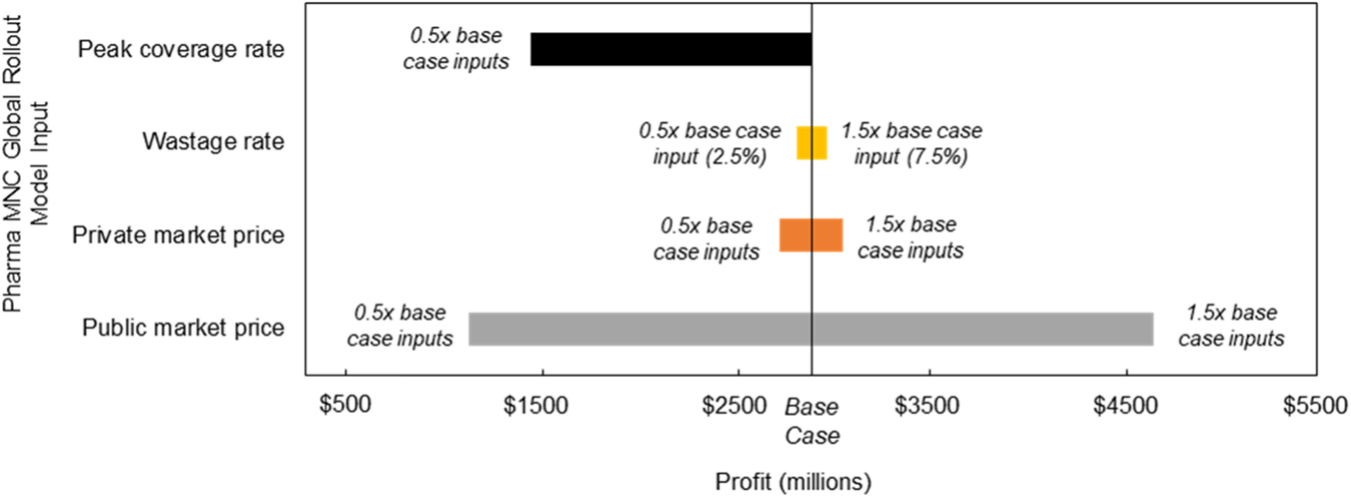
Profit sensitivity analysis. Sensitivity of forecasted profit to select model inputs for the infant immunization program at year 12 for the Pharma MNC Global Rollout scenario.

The sensitivity of the forecasted profit at year 12 for the Pharma MNC Global Rollout, infant immunization program scenario to changes in price (public and private markets), wastage rate, and peak coverage rate model inputs were assessed (Fig. 5). The model is more sensitive to changes in public market price than private market price and exhibits low sensitivity to similar changes in the wastage rate (as a % of baseline values). The impact of changes in peak coverage rate on profit is directly proportional (i.e., 50% reduction in peak coverage across all countries leads to 50% reduction in total annual profit).

### Net present value (NPV) analysis

The viability of the commercial market for Strep A vaccine development depends on the potential return on investment required to bring a Strep A vaccine to market. Such investments include expenses for conducting clinical trials, manufacturing scale-up, WHO prequalification, licensure, post-marketing activities as well as building manufacturing facilities. For both Pharma MNC and DCVM scenarios, vaccine procurement through UNICEF was assumed to support purchase for the 49 countries eligible for Gavi support (based on 2021 eligibility). The Gavi-eligible countries designated in the current model include 31 LICs and 18 LMICs. Countries designated by Gavi as in the “accelerated transition” or “fully self-financing” phases were not considered Gavi-eligible in the current model to account for the time between current eligibility status and the market launch of the hypothetical vaccine in 2033. In addition to the country where the DCVM is located, these companies also typically supply to surrounding countries though interviews with DCVM representatives and vaccine market experts suggest DCVMs are increasingly pursuing higher-income target markets. For the purpose of this scenario, the DCVM target markets were assumed to be LICs/Gavi-eligible countries, LMICs and UMICs starting in 2033, and HICs starting in 2038.

To estimate the investment return for the development of a Strep A vaccine, the net-present value (NPV) was calculated using the risk-adjusted investment cost (also referred to as attrition-adjusted investment cost), incorporating the cumulative cost of failures at various stages of development^18,19,22,30–34^, along with a discount rate that approximates the cost of capital for pharmaceutical companies^17–19,30,33,35^. The estimated investment return for the development of a Strep A vaccine is summarized in Table 1. For the Pharma MNC Global Rollout scenario, the NPV at year 12 is ~$2.5 billion and $2.0 billion for the infant and child immunization program scenarios, respectively, assuming a risk-adjusted total development and manufacturing investment of $979 million (see Methods for detailed breakdown) and a discount rate of 10%^17,18,30^. For the DCVM scenario, the year 12 NPV is approximately $310 million and $210 million for the infant and child immunization programs, respectively, assuming a risk-adjusted total development and manufacturing investment of $372 million (see Methods for detailed breakdown) and a discount rate of 15%. The higher assumed discount rate for the DCVM aligns with the midpoint of a discount rate range reported previously^35^ and with observations that smaller biopharmaceutical companies generally have higher costs of capital than Pharma MNCs^18,19^. In addition to having a higher NPV, the Pharma MNC Global Rollout scenario also has a greater gross profit margin (profit margin before selling, general & administrative costs) than the DCVM scenario. This is due to the higher proportion of demand from HIC markets (with associated higher prices and profit margin) in the Pharma MNC Global Rollout scenario. However, a positive NPV is still estimated in a scenario wherein there is no demand from HIC markets (see Supplementary Table 7). The gross profit margin at year 12 for the child immunization program is slightly higher than for the infant immunization program in both the Pharma MNC Global Rollout and DCVM Staged Rollout scenarios, due to the relative proportion of private market versus public market at year 12, which is greater for the child program than for the infant program (see Fig. 4). The NPV of the Pharma MNC Staged Rollout scenario is $1.3 billion and $720 million for the infant and child programs, respectively (see Supplementary Table 7). In the Optimistic Timeline scenario for the Pharma MNC Global Rollout scenario, the year 12 NPV for the infant program scenario increases from $2.5 billion to $6.7 billion. In the Conservative Timeline scenario, the NPV decreases from $2.5 billion to $1.5 billion (see Supplementary Table 8).

**Table 1:** Gross profit margin and net present value (NPV) at year 12.

	Infant program scenario year 12 financial analysis		Child program scenario year 12 financial analysis	
Investment scenario	NPV (millions USD)	Gross profit margin	NPV (millions USD)	Gross profit margin
Pharma MNC global rollout	$2460	75%	$1990	77%
DCVM staged rollout	$307	64%	$210	66%

Year 12 gross profit margin and NPV for pharmaceutical multinational corporation (Pharma MNC) and developing country vaccine manufacturer (DCVM) scenarios.

**Fig. 6:**
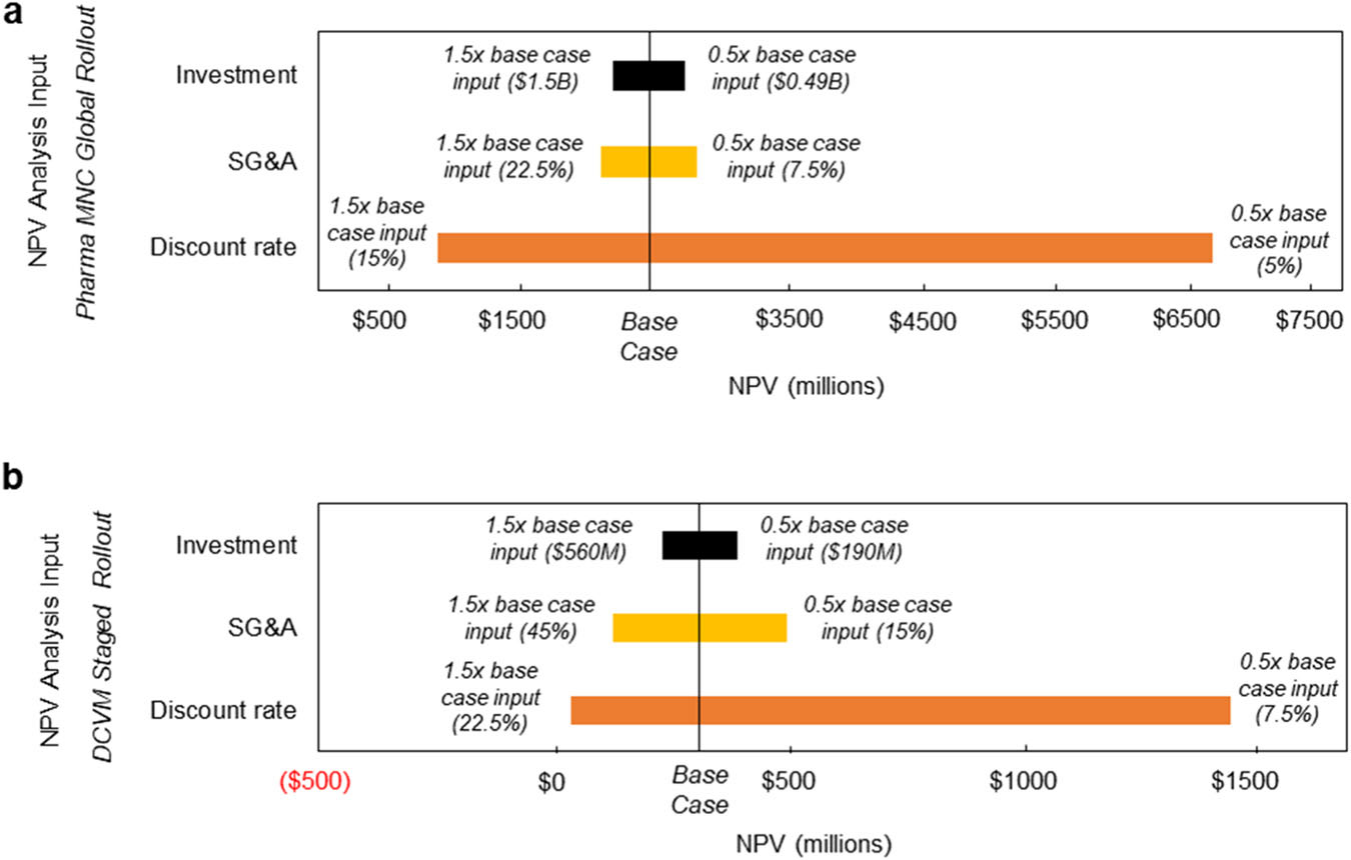
Net present value (NPV) sensitivity analysis. Sensitivity of forecasted NPV to financial assumptions for the **a** Pharma MNC Global Rollout and **b** DCVM Staged Rollout infant immunization program scenarios at year 12.

The sensitivity of the year 12 NPV for the Pharma MNC Global Rollout and DCVM Staged Rollout infant immunization program scenarios to assumptions for discount rate (estimated at 10% for Pharma MNC scenario; 15% for DCVM scenario), selling, general & administrative costs (estimated to be 15% and 30% of revenues for the Pharma MNC and DCVM scenario, respectively), as well as total investment, was assessed (Fig. 6). The NPV analysis is highly sensitive to changes in the discount rate assumption. It should also be noted that individual companies have their own proprietary discount rates for NPV analyses and thus the NPV results in this paper should be interpreted as approximations of potential investment return that would change if discount rates were raised or lowered.

Finally, though a single hypothetical Strep A vaccine has been modeled, there is a possibility that one or more competitor Strep A vaccines could enter the market following launch of the hypothetical first Strep A vaccine. To determine the impact such competitive events may have on the NPV forecast, multiple competitive entry scenarios framed around a loss of profit (25% or 50%) beginning 3, 5, or 7 years after market entry of the hypothetical Strep A vaccine were modeled. A competitive entry is likely to impact not just market share (i.e., revenue) but also market price; therefore, loss in profit is used to determine the potential effect of a competitive entry on the NPV. The year 12 NPV is favorable (i.e., positive in value) for all competitive entry scenarios and market scenarios. A substantial competitive event would be required to render the Strep A vaccine development market unviable commercially, for example, a complete loss of all profit by year 3 in the case of the Pharma MNC Global Rollout scenario.

## Discussion

The outcomes of this study suggest there is a viable commercial market for private sector investment in Strep A vaccine development. The investment return analysis found a positive NPV for investment in Strep A vaccine development across multiple scenarios, including: (i) different types of vaccine developers (i.e., global and staged rollout by a Pharma MNC, staged rollout by a DCVM); (ii) different target populations for the vaccine (i.e., infant or child immunization program scenarios); and (iii) several competitive event scenarios. It is hoped that these results can raise awareness of the potential commercial viability of the Strep A vaccine market and serve as a resource for companies assessing potential investment in Strep A vaccine R&D.

Currently, only two active Strep A vaccine product development programs are being led by private sector companies—the GSK Vaccines Institute for Global Health (Italy) and Vaxcyte (USA)—reflecting the historical lack of perceived commercial potential for a Strep A vaccine^6^. In addition to a lack of investment from pharmaceutical companies, the public and not-for-profit sectors are not prioritizing Strep A vaccine development, exemplified by the substantial underfunding of Strep A/RHD R&D relative to its disease burden^9^. Further, developers are not only influenced by the potential market size for a vaccine but also feasibility of meeting such demand. The prospect of Strep A vaccine development is negatively affected by scientific and technical challenges as well as regulatory uncertainty. While several Strep A vaccine candidates have showed efficacy in various preclinical models, no candidate has demonstrated efficacy in humans^6,36^. Further, the absence of putative vaccine-induced immune correlate(s) of protection, the impact of high strain diversity on breadth of vaccine efficacy and the lack of high-throughput, standardized assays to measure Strep A vaccine-induced immune responses are additional obstacles to a straightforward R&D process. The regulatory pathway toward licensure of a Strep A vaccine is unclear, as regulators have yet to signal which specific endpoints, target populations, and study design considerations they will expect developers to follow. Further, safety concerns present another challenge for developers to navigate. Though the decades-long ban (due to historical safety concerns) on clinical testing of Strep A vaccines was lifted over 15 years ago^7^, there will be considerable scrutiny around adequate safety testing and monitoring during clinical development (and in post-marketing studies), and this may be seen as an additional factor compounding the lack of regulatory clarity and affecting the private sector’s perception of the feasibility of Strep A vaccine R&D.

Though the current study suggests there would be a commercially viable market for Strep A vaccines in the future, the results rest on the assumption that in-country decision-makers, supranational agencies and normative bodies, and public health funders across the globe will increasingly recognize the value of Strep A vaccination by the time a Strep A vaccine is ready for rollout. More specifically, the current model results assume that countries around the world will collectively adopt (over a period of 15 years from the time of first market launch for the base case timeline scenario) a Strep A vaccine into their respective NIPs and gradually ramp up Strep A vaccine delivery over a 10-year period until it reaches a level of uptake equivalent to the countries’ respective coverage level for DTP3 (assuming Strep A vaccine is offered to infants) or MCV2 (should countries instead offer Strep A vaccine to school-age children aged 4–7). Achieving these coverage levels will depend on several implementation factors including procuring adequate vaccine supply (which for many LICs and LMICs will be aided by UNICEF/Gavi support), efficient distribution and storage across healthcare settings, availability of sufficient numbers of trained staff for vaccine administration as well as public health campaigns and community engagement efforts to raise awareness of the availability and benefit of Strep A vaccination and to counteract any vaccine misinformation/hesitancy.

RHD burden is considered one of the main drivers for the development and adoption of a Strep A vaccine due to the high morbidity and mortality associated with the disease. As such, RHD burden was used to help estimate likelihood of adoption on a per-country basis in the Timing of Introduction Analysis. However, pharyngitis and impetigo have been strategically targeted as clinical endpoints of a Strep A vaccine for feasibility (see Methods, Table 2), and while there is clinical data showing that prevention of pharyngitis and other acute Strep A infections reduces the incidence of immune-mediated sequalae including ARF^37^, pharyngitis, and impetigo are both assumed to be primary intermediates on the causal pathway to ARF and RHD, and it is understood that more longitudinal data will be required in the future to strengthen this assumption, understand population vaccine impact and inform policymaking^38,39^.

**Table 2:** Hypothetical Strep A vaccine target product profile (TPP).

Parameter	Characteristics
Vaccine type	Multivalent recombinant vaccine (e.g., protein subunits, group A carbohydrate), adjuvant
Indication(s)	Prevention of Strep A-related pharyngitis and superficial skin infection
Target population(s)	Infants (<1 year) or young children (between ages 4 and 7)
Primary regimen	3 doses administered on days ~0, 30, and 90; no booster
Efficacy*	80% protection against non-severe, non-invasive confirmed Strep A disease
Strain coverage*	At least 90% of the current disease-causing isolates from region targeted
Safety*	Favorable safety and reactogenicity profile, no cross-reactivity with kidney/cardiac tissue, and non-interference upon co-administration with recommended other vaccines
Route of administration	Intramuscular injection
Durability^†^	At least 10 years
Adjuvant	Aluminum hydroxide
Stability^†^	Stable at 2–8 °C with a shelf-life of at least 24 months
Presentation^†^	1-dose/vial or pre-filled syringe, liquid

Hypothetical Strep A vaccine Target Product Profile (TPP), based on selected parameters from the WHO preferred product characteristics (PPCs) for a Strep A vaccine and characteristics of the most advanced Strep A vaccine candidates^6,7^.

*As per the WHO preferred product characteristics for Group A Strep vaccines.

^†^Parameter not included in the WHO preferred product characteristics.

Vaccine demand from HICs will play a significant role in the commercial viability and attractiveness of Strep A vaccine development to Pharma MNC developers, as HICs yield most of the of revenue and profits due to higher vaccine prices in these markets. While the need for a Strep A vaccine is more obvious in LICs and LMICs where the burden of ARF and RHD is relatively higher, there is substantial burden of other Strep A diseases in HICs, including pharyngitis, impetigo, and scarlet fever^1^. These diseases do not cause substantial mortality like RHD, but they lead to significant antibiotic use and other economic costs^25^ and an underappreciated broader societal burden^26^. Indeed, a member of the US CDC’s Advisory Committee on Immunization Practices (ACIP) stated in 2018 that an effective and safe Strep A vaccine would be “recommended universally for children” in the US^40^. However, even if the value of Strep A vaccination is not recognized immediately by HICs, resulting in a delay of several years before the first HICs begin to adopt the vaccine, our results still demonstrate a positive NPV for Pharma MNC developer investment in Strep A vaccine R&D. In fact, if the contribution of demand from HICs is totally removed, the NPV across developer and target population scenarios remains positive, demonstrating that rollout in non-HICs can still be commercially viable for Pharma MNCs and DCVMs. However, the existence of a ‘dual market’ for Strep A vaccines, in which there would be rollout in HICs as well as LICs and middle-income countries (MICs) would enhance the sustainability and robustness of the overall Strep A vaccine market by enabling higher total volumes of demand as well as a higher average price and hence profit (due to the relatively higher vaccine prices in HICs) for developers. In fact, an active market in HICs will help to offset any risk associated with slower than expected uptake in LICs and MICs. Finally, it will be important for future studies to estimate the demand and investment return associated with adult target populations in which the indication for a Strep A vaccine will be to prevent invasive diseases like cellulitis which drive significant health and economic burden in adults including in HICs^41–43^. An adult market for Strep A vaccination would only add to the potential commercial return on investment in Strep A vaccine R&D by Pharma MNC developers.

In addition to the current study as well as others aimed at substantiating the burden of Strep A disease and the cost-effectiveness and public health and broader societal impact of Strep A vaccination^25–27^, the field will benefit from stronger funding from global health and government funders to de-risk Strep A vaccine development and increase the probability of Strep A vaccine programs being prioritized by the companies with the greatest potential to bring a product to market. Vaccine developers and manufacturers are considered the primary audience for this study, but given the role of governments, international organizations, and global health funders in catalyzing private sector investment in vaccine R&D, these other stakeholder types are considered as a secondary audience. While it is encouraging that the model indicated various scenarios with a positive NPV for Pharma MNC or DCVM investment in Strep A vaccine clinical development, manufacturing, licensure, and post-marketing activities, it is not a foregone conclusion that a company would invest in Strep A vaccine development given the potential that other investment opportunities (e.g., vaccines for other infectious diseases or medicines for different therapy areas) may present more attractive business cases driven by even higher NPVs. Thus, the availability of global health funding to subsidize parts of the development journey (e.g., funding the cost of one or more clinical trials or building manufacturing capacity), which would increase the NPV, represents an important mechanism to de-risk and further incentivize industry prioritization of Strep A vaccine development. Global health funding could also mitigate the risk of a company placing a higher price on a Strep A vaccine to recoup the larger investment required without supplementary funding^44^. In addition to stimulating greater industry investment, this funding could also be directed in a manner that helps build regional or local capacity (e.g., clinical trial or manufacturing infrastructure) that could contribute to global public health objectives beyond the Strep A vaccine program. Finally, it should be noted that global health funding may entail certain obligations (e.g., related to program governance, vaccine access provisions, intellectual property) that companies could perceive as burdensome or misaligned with business imperatives^45^. Thus, it will be important for funding arrangements to optimize the balance of benefits (i.e., non-dilutive funding) and perceived costs to achieve greater industry prioritization of Strep A vaccine development.

Limitations of the current study center on the degree to which the model accurately predicts adoption of a Strep A vaccine for market entry in specific countries. The vaccine demand forecast component of the model is driven by each country’s level of Strep A burden (using RHD incidence as a proxy), track record in introducing recent vaccines (Hib, pneumococcal, and rotavirus vaccines), and overall vaccination infrastructure (DTP3 coverage). Outputs were then manually adjusted based on interviews with biopharmaceutical industry experts to reflect vaccine industry commercial market dynamics, and in-country vaccine decision-makers to reflect signals of prioritization of Strep A disease management and/or Strep A vaccine R&D (e.g., Australia’s investment in The Australian Strep A Vaccine Initiative [ASAVI] which is supporting efforts in vaccine development, disease surveillance, and community engagement)^24^. These interviews, in alignment with previous characterizations in the literature, revealed that Strep A is an underappreciated public health threat—particularly in low- and middle-income countries—in large part due to a lack of disease registries and active surveillance systems as well as underreporting of cases^7^. However, given the known high global burden of Strep A disease, it is likely that the Strep A FVVA Report as well as continued advocacy and programmatic efforts through groups such as SAVAC as well as others like the WHO will increase demand for a Strep A vaccine by the time one is ready for the market. Future work aimed at more precisely forecasting country-level demand will be important to undertake, especially once more data on Strep A vaccine candidates (e.g., human efficacy results), as well as more granular epidemiology and burden data on all Strep A diseases, are available.

In summary, through a novel Strep A vaccine global demand and return on investment forecast model, the current study demonstrates there is a viable commercial market for Strep A vaccine development. It is hoped that the model and its outputs lead to greater prioritization of Strep A vaccine development by informing investment decision-making and driving engagement among vaccine manufacturers, whether multinational pharmaceutical corporations, smaller biotech companies, or developing country vaccine manufacturers, as well as global health funders who may catalyze industry investment.

## Methods

### Overview

Since current Strep A vaccine candidates have not yet progressed beyond Phase 1 clinical studies, vaccine product and development features including clinical endpoints, potential efficacy, durability, manufacturing feasibility and cost per dose are unknown. Potential adoption into vaccination programs and uptake through the private market are also an uncertainty, which is further complicated by the lack of country-level surveillance and epidemiological data on Strep A diseases^7^. Therefore, the Strep A vaccine global demand and return on investment forecast model leverages traditional demand and return on investment methodological approaches (described and referenced herein), a landscape assessment of Strep A vaccines in development^6^, information from proxy vaccines sourced from the WHO vaccine-preventable diseases monitoring system^20^, the Market Information for Access to Vaccines database (MI4A)^21^, as well interviews with infectious disease and vaccine experts, global health funders, in-country vaccine decision-makers and representatives from Pharma MNCs and DCVMs. The following is an overview of the key inputs and assumptions used in the demand and investment forecast model.

### Hypothetical target product profile

The forecast model is informed by a hypothetical Strep A vaccine Target Product Profile (TPP, Table 2) which is based on the WHO Preferred Product Characteristics (PPCs) for a Strep A vaccine^7^, as well as common characteristics of the most advanced current Strep A vaccine candidates^6^. The model assumes that the vaccine will protect against Strep A-induced pharyngitis and superficial skin infection (i.e. impetigo), diseases that will likely serve as the clinical endpoints for efficacy trials. The analysis includes two different target population modeling scenarios chosen based on the age of peak pharyngitis incidence (5–15 years of age^1^) and practical considerations related to uptake: infants (less than 1 year) or young children (4–7 years).

### Market launch and timing of introduction

Based on the status of Strep A vaccine candidates currently in early clinical development, and assuming the developer of the Strep A vaccine would seek WHO prequalification and SAGE/NITAG recommendation, it is estimated that a hypothetical vaccine would reach the market in 2033 (see Financial Analysis section for details). It is assumed that DCVMs and, in a secondary, ‘Staged Rollout’ scenario, Pharma MNCs may engage in a staged rollout wherein the vaccine is introduced in lower-income countries with a greater proportion of Strep A burden before market launch in high-income countries (HICs) 5 years later (i.e., in 2038). In the Pharma MNC Global Rollout scenario, HICs are assumed to begin adopting the vaccine in 2033 with no delay compared to lower-income countries.

It is assumed that countries would introduce the vaccine in their national immunization program (NIP) over a period of up to 15 years after market launch, based on historical examples^20^. The specific year over the 15-year period at which each country included in the model is estimated to introduce the vaccine in their NIP was determined via a scoring system based on country-specific data across the following 3 parameters: (1) RHD burden: in the absence of sufficient country-level surveillance of Strep A-induced pharyngitis and impetigo, and due to the expected public health focus on the high RHD-associated morbidity and mortality, RHD incidence rate was used to assess a country’s Strep A burden (age-standardized, combined males and females, rate/100,000)^2^; (2) vaccine adoption history: the extent to which a country has adopted contemporary WHO-recommended vaccines into its NIP by 2020 (i.e., Hib, pneumococcal, and rotavirus vaccines) provided a sense of a country’s track record of introducing new vaccines; and (3) vaccine delivery infrastructure: a country’s ability to support vaccine uptake under its current vaccination schedule was assessed by using the country’s coverage rate of the third dose of diphtheria, tetanus, and pertussis vaccine (DTP3) (also used by Gavi as a benchmark to assess the implementation capacity of a potential Gavi-eligible country)^46^. The following weighting was used for each parameter: (1) RHD burden: 20%; (2) vaccine adoption history: 40%; and (3) vaccine delivery infrastructure: 40%. RHD burden was given a relatively lower weighting because RHD burden does not reflect the burden of all Strep A diseases. As confirmed in interviews with in-country vaccine decision makers, countries with low RHD burden may still have interest in the vaccine to prevent acute infection and its proximate disease manifestations (i.e., pharyngitis and impetigo).

Any other evidence collected through subject matter expert interviews or the literature that indicated a specific country’s interest in adopting a Strep A vaccine, such as government prioritization and market dynamics considerations (e.g., status of a country as a major biopharmaceutical target market) is incorporated into the timing of introduction modeling via a qualitative adjustment. For example, it is assumed that for Gavi-eligible countries, the process of qualifying for Gavi support and UNICEF procurement commitment would require an additional 2 years beyond regulatory licensure. Thus, the first year of market entry for Gavi countries was assumed to be 2035 rather than 2033. In-country vaccine decision-makers were interviewed from a collection of countries across various regions and income levels, including Mozambique, Nepal, Haiti, India, Bangladesh, Papua New Guinea, Thailand, Fiji, South Africa, Germany, France, UK, Australia, and New Zealand.

Two additional timeline scenarios were tested: an Optimistic Timeline scenario and a Conservative Timeline scenario. In the Optimistic Timeline scenario, it is assumed that development timelines as well as NIP introduction may accelerate due to the precedent set by COVID-19 vaccine development and rollout (8 years for development timelines vs. 11 in the base case scenario and 8 years for maximum spread of years of introduction vs. 15 in the base case scenario). In the Conservative Timeline scenario, the maximum number of years over which countries introduce the vaccine into their NIPs adoption aligns with historical examples of vaccines that took longer for widespread adoption, such as HepB (20 years vs. 15 in the base case scenario).

### Global population forecast

The forecast model includes all countries that were presented in the UN Population data estimates^47^, WHO vaccine coverage rate databases^20^ and a secondary analysis of the GBD 2017 rheumatic heart disease (RHD) database^2^. An equal number of people at each age within the 5-year buckets presented in the UN Population Database is assumed.

### Market segments

Markets in the forecast are defined by public vs. private markets (i.e., NIP or private healthcare provider, respectively) and country income level. Assumptions related to private and public market uptake for each country income segment are described below. Each country is segmented by region and country income level based on the 2020 country income-level classification criteria from the World Bank^48^. Country income levels include low-income countries (LICs), lower middle-income countries (LMICs), upper middle-income countries (UMICs) and high-income countries (HICs).

### Private market coverage rate

It is assumed that the vaccine will be available via the private market to infants and children aged 0–6 years upon the first year of market entry and for the entire modeling period. The estimated total available size of the private market is 5% of the population for LICs, 10% of the population in LMICs, 20% of the population in UMICs, and 35% of the population in HICs. The size of the private market is likely to decrease once the vaccine becomes available in the public market when individuals may get vaccinated at lower or no personal expense except in LICs where the small proportion of those who access the vaccine privately may remain constant after public vaccine introduction. The size of the private market is therefore adjusted to 5% LMICs, 10% in UMICs, and 0% of the population in HICs once a country introduces the vaccine into its NIP. Peak coverage rates in the private market (i.e., proportion of total available private market size that takes the Strep A vaccine) were estimated to be 30% for LICs and LMICs, 15% for UMICs, and 10% for HICs. The peak coverage rate in the private market is assumed to be reached 3 years after market entry and follow a linear increase from zero. These private market assumptions were informed through interviews with vaccine industry experts and several of the DCVMs that serve these markets.

### Public market coverage rate

The forecast model uses actual country-level vaccine coverage rates of proxy vaccines to estimate the potential uptake of a Strep A vaccine in the public market. Coverage rates of established proxy vaccines with long-standing surveillance results, and for which peak rates are assumed to have been reached, are used. DTP3 2019 coverage rate is used as the peak coverage rate for the infant immunization program. DTP3 is used rather than DTP1 to avoid over-estimation of potential uptake. The second dose of each country’s measles-containing vaccine (MCV2) 2019 coverage rate is used as the peak coverage rate for the child immunization program in countries that have an MCV childhood immunization program (i.e., ages 4–7)^20^. For countries without a school-age immunization program, a maximum coverage rate of 50% is assumed. The sensitivity of the model to the public market coverage rate estimates was assessed: the change in profit at year 12 is reported for the infant immunization program of the Pharma MNC Global Rollout and DCVM Staged Rollout scenarios if public market coverage rates are 50% of what is assumed in the base case (i.e., 50% of DTP3 2019 coverage rate).

The peak coverage rate is assumed to be reached 10 years after the year of introduction with a linear increase from zero to peak coverage over those 10 years. In the Optimistic and Conservative Timeline scenarios, it is assumed that ramp-up to peak coverage takes place over 5 years and 15 years, respectively. This assumption is based on WHO vaccine surveillance reporting for infant and childhood vaccines introduced in public markets across country income levels within the last 20 years, including PCV3, RotaC, MCV2, and second dose of varicella (greater than 10 years to reach peak coverage)^20,49^.

### Vaccine presentation and cost

It is assumed that single-dose vaccine vials will be used in all markets in LIC, LMIC, and UMICs and pre-filled syringes will be used in the HIC public market as well as the private markets of UMIC and HICs. The vaccine presentation assumptions for the public market are based on historical vaccine procurement data from the WHO MI4A database using PCV13, human papillomavirus 2/4 (HPV 2/4) and rotavirus (Rota) as proxy vaccines^21^ with the caveat that multi-dose vials could potentially be more beneficial in some vaccination scenarios such as campaigns in school settings when wastage rate is less of a concern. Vaccine presentation assumptions for the private markets are based on the assumption that HIC and UMIC private markets would procure pre-filled syringes due to ease of vaccine administration, while LIC and LMIC private markets would procure single-dose vials rather than pre-filled syringes for cost-effectiveness reasons. A wastage rate for both vaccine presentations of 5% is assumed^28,29^. The sensitivity of the model to the wastage rate assumption was assessed: the change in profit at year 12 is reported for the infant immunization program of the Pharma MNC Global Rollout and DCVM Staged Rollout scenarios if wastage rate is 50% and 150% of what is assumed in the base case (i.e., 2.5% and 7.5% vs. 5%).

The cost per dose of vaccine (or cost of goods sold, COGS) depends on the technical complexity of the vaccine, presentation, as well as economies of scale that could be achieved through existing multivalent protein manufacturing processes established for other vaccines. Given that cost data on vaccines is proprietary information, information from a published study detailing costs of the polio vaccine (IPV)—with an adjustment to account for the relatively more complex manufacturing requirements expected for a Strep A vaccine—was used to estimate COGS^29^. The Strep A vaccine COGS/dose in USD is estimated to be $3.24/dose for a 1-dose vial and $3.18/dose for a pre-filled syringe. These cost estimates were validated during interviews with industry experts, including representatives from DCVMs. Vaccine implementation and delivery costs were not included in the cost analyses as these costs are typically incurred by local health system authorities (potentially subsidized by global health funders) and not the vaccine manufacturers.

### Vaccine price

The price per dose used in the model is based on price data for PCV13 from the MI4A: Market Information for Access to Vaccines database across different country income-level groups (Table 3)^21^. The PCV13 vaccine was used as a proxy to estimate the price of the Strep A vaccine given the similarity in complexity of the two vaccines (10/13 valent, inclusion of multiple CRM197 carrier proteins) and the extent to which pricing data is available for this vaccine. The WHO MI4A Database was used to calculate an average price for the PCV13 vaccine in 1-dose vials and pre-filled syringe formulations across the HIC, UMIC, LMIC, LIC, and Gavi-eligible markets. Given that the price of PCV13 has declined significantly over the years and because a Strep A vaccine may not command the premium pricing of PCV13 in early years, the 2019 PCV13 prices are used as proxies. These prices are also in the range of what UNICEF, PAHO, self-procuring MICs, and HICs pay for the HPV 2/4 vaccines^21^. The sensitivity of the model to the price estimates was assessed: the change in profit at year 12 is reported for the infant immunization program of the Pharma MNC Global Rollout and DCVM Staged Rollout scenarios if prices are 50% or 150% of those assumed in the base case (e.g., $26.98 and $107.90 vs. $53.95 for the pre-filled syringe HIC price).

**Table 3:** Vaccine presentation and price assumptions.

Country income-level group	Public market vaccine price/dose (USD) and presentation	Private market vaccine price/dose (USD) and presentation
High-income	$53.95 (pre-filled syringe)	$53.95 (pre-filled syringe)
Upper middle-income	$13.47 (1-dose vial)	$28.39 (pre-filled syringe)
Lower middle-income	$6.18 (1-dose vial)	$6.18 (1-dose vial)
Low-income and Gavi-eligible	$3.40 (1-dose vial)	$3.40 (1-dose vial)

Price per dose and vaccine dose presentation assumptions in public vs. private markets across country income-level groups.

### Financial analysis

An investment return analysis was completed to estimate the financial attractiveness of investment in the development, commercialization, and production of a Strep A vaccine for Pharma MNCs and DCVMs. Different estimates of total investment costs and other financial inputs are used for the Pharma MNC and DCVM scenarios. For the Pharma MNC scenario, both staged and global rollout scenarios are considered, while only a staged rollout is considered in the DCVM scenario. In the Pharma MNC Global Rollout scenario, the vaccine first enters the market across all country income-level groups in 2033. In the DCVM and Pharma MNC Staged Rollout scenario, the vaccine first enters the market in LICs, LMICs, and UMICs in 2033 and HICs in 2038.

The total R&D investment for the Pharma MNC (Global and Staged Rollout) and DCVM Staged Rollout scenarios are assumed to be $528 million and $185 million, respectively, including potential costs associated with preclinical studies, clinical trials, process development and scale-up, licensure and WHO prequalification, building manufacturing capacity as well as post-marketing activities (Table 4). All cost estimates are based on literature for typical vaccine R&D investments^22,34,44,50^ as well as expert interviews. Details about trial design specific to a Strep A vaccine are uncertain at this point given that no Strep A vaccine has yet entered a Phase 2 trial and that regulatory requirements for Phase 2 or 3 trials are unclear. To account for potential failures along the path to licensure, these investment estimates are risk-adjusted according to the probability of success (POS) for advancing from a given stage of development to the next. The POS for transitioning from each stage of development was estimated using historical vaccine development POS rates with modifications based on input from expert interviews: 60% POS for preclinical development, 75% POS for Phase 1, 40% POS for Phase 2, 80% POS for Phase 3 and 90% POS for all post-clinical development activities^22,34,50–52^. Total risk-adjusted investment costs were calculated using a formula that weights the estimated investment at each stage of development according to the estimated POS for that stage (Equation 1)^52^.

**Table 4:** Summary of investment costs.

	Pharma MNC investment (millions, USD)	DCVM investment (millions, USD)
Preclinical development	$26	$8
Phase 1	$20	$10
Phase 2	$40	$25
Phase 3	$200	$70
Process development	$12	$10
Regulatory activities	$5	$2
Building manufacturing capacity	$150	$40
Post-marketing activities	$75	$20
Total	$528	$185
Risk-adjusted total	$979	$372

Investment costs for each stage of Strep A vaccine research and development.

Equation 1. Risk-Adjustment of R&D Costs Equation. Calculation of risk-adjusted R&D costs, accounting for probability of success at each stage of development (POS_*n*_) and investment at each stage of development (Cost_*n*_*)*.

$${Total\; Risk{\hbox{-}}Adjusted\; Investment}=\frac{{{Cost}}_{1}\times {{POS}}_{1}+{{Cost}}_{2}\times {{POS}}_{2}+{{Cost}}_{3}\times {{POS}}_{3}+\ldots }{{{POS}}_{1}\times {{POS}}_{2}\times {{POS}}_{3}\times \ldots }$$

It is assumed that Phases 1, 2 and manufacturing scale-up would be completed in 2025, Phase 3 and lot-to-lot consistency and bridging studies would conclude in 2030, and regulatory, WHO prequalification and NITAG recommendations would be completed in year 2033 (year of market entry). In the Optimistic Timeline scenario, these development timelines are assumed to be accelerated to 2023, 2026, and 2030, respectively.

A standard net present value (NPV) analysis using the risk-adjusted capital investments (Table 4), operating profit, and discount rates reflecting industry-specific cost of capital was used to estimate the value of the development project for each developer scenario and timeline scenario. The discount rate was applied to projected annual cash flows over 12 years to estimate the value of the project across scenarios in today’s dollars (Equation 2). Such analyses typically are used to estimate investment return of a capital investment project and, when used in conjunction with sensitivity analyses, can provide an understanding of the impact of key assumptions on the potential profitability of the business model^16,17^.

Equation 2. Net Present Value Equation. Net present value (NPV), where the (Net Cash Flow)_*t*_ is the net cash flow over one year (based on operating profit and risk-adjusted capital investments), the Discount Rate is the risk-adjusted discount rate applied to estimate the value of future cash flows in today’s dollars, and *t* is the number of years over which the NPV is calculated. NPV was calculated over 12 years in the current analysis.

$${NPV}=\mathop{\sum }\limits_{t=0}^{12}\frac{{({Net}\,{Cash}\,{Flow})}_{t}}{{(1+{Discount}\,{Rate})}^{t}}$$

The NPV for the Pharma MNC scenario was calculated using a discount rate of 10%, which reflects the weighted average cost of capital (WACC) for companies in this industry and which aligns with discount rates used in other studies.^17,18,30^ The NPV for the DCVM scenario was calculated using a discount rate of 15%. The higher assumed discount rate for the DCVM aligns with the midpoint of a discount rate range reported previously^35^ and with observations that smaller biopharmaceutical companies generally have higher costs of capital than Pharma MNCs^18,19^. It is important to note that individual companies have their own proprietary discount rates for NPV analyses and thus the NPV results in this paper should be interpreted as approximations of potential investment return that would change if discount rates were raised or lowered. Operating profits were calculated by subtracting selling, general and administrative costs (SG&A) from gross profit. Annual SG&A costs were calculated as a percent of revenues, varying by developer type (15% for Pharma MNCs and 30% for DCVMs)^53–55^. NPV was then calculated under various competitive event scenarios in which profits were assumed to be either 25% or 50% lower beginning either 3, 5, or 7 years after market entry.

The sensitivity of the NPV analysis to the discount rate and SG&A inputs were assessed: the change in the NPV at year 12 is reported for the infant immunization program of the Pharma MNC Global Rollout and DCVM Staged Rollout scenarios if inputs are 50% or 150% of those assumed in the base case (e.g., discount rate of 5% and 15% vs. 10% for the Pharma MNC Global Rollout scenario).

### Reporting summary

Further information on research design is available in the Nature Research Reporting Summary linked to this article.

### Supplementary information

Supplementary Materials

REPORTING SUMMARY

## Data Availability

The data that support the findings of this study are available from the corresponding author upon request.
