# Supplementary material for: A Strep A vaccine global demand and return on investment forecast to inform industry research and development prioritization
Source: NPJ Vaccines. 2023 Aug 9;8:113. doi: 10.1038/s41541-023-00690-2 (PMC10412591; doi:10.1038/s41541-023-00690-2)
Supplement: Supplementary file 1 — Supplementary Materials [file 41541_2023_690_MOESM1_ESM.pdf]

A Strep A vaccine demand and return on investment forecast to inform industry R&D prioritization decisions

Supplementary Materials

Donald R Walkinshaw[1,*], Meghan EE Wright[1], Marni Williams[1], Tanya MF Scarapicchia[1], Jean-Louis Excler[2], Ryan E Wiley[1], Anne E Mullin[1]

[1] Shift Health, Toronto, Canada
[2] International Vaccine Institute, Seoul, Republic of Korea

* Corresponding Author: dwalkinshaw@shifthealth.com

Timing of Introduction Country-by-Country Results

Supplementary Table 1. Year of Introduction Scoring System. Country-level data across 3 dimensions was used to estimate the time within which a country would be expected to adopt the Strep A vaccine in its national immunization program (NIP): RHD disease burden (age-standardized incidence rate), vaccine adoption history (based on adoption of Haemophilus influenzae type b [Hib], rotavirus [Rota] and pneumococcal conjugate vaccine [PCV]) and vaccine delivery infrastructure (based on 2019 DTP3 coverage). For each dimension, countries were assigned to one of three groups using the criteria outlined here. Each group was assigned a value from an integer point scale of 0, 2 and 4, such that the maximum total score is 4 for RHD Disease Burden, 12 for Vaccine Adoption History and 4 for Vaccine Delivery Infrastructure.

		Group 1 (Assigned Value of 0)	Group 2 (Assigned Value of 2)	Group 3 (Assigned Value of 4)	Maximum Total Score per Dimension
**RHD Disease Burden[1]**		Incidence rate of >24.4	Incidence rate between 12.7 and 24.4	Age standardized RHD incidence rate of <12.7	4
**Vaccine Adoption History[2]**	**Hib**	Implemented in NIP and coverage >0% reported	Planned implementation; Included in NIP but coverage not reported; Administered in special circumstances/regions	Not implemented in NIP	12
	**Rota**	"	"	"	
	**PCV2**	"	"	"	
**Vaccine Delivery Infrastructure[2,3]**		DTP3 reported coverage >90%	DTP3 reported coverage between 70 and 89%	DTP3 reported coverage of <70%	4
				**Maximum Total Score Per Country**	**20**

[1] Countries were divided into 3 bins of equal distribution from low to high RHD incidence rate (new cases per 100,000). Data source: https://www.ahajournals.org/doi/full/10.1161/JAHA.122.025284

[2] Information current as of 2019 and based on WHO vaccine-preventable diseases: monitoring system: https://immunizationdata.who.int/

[3] Coverage thresholds were selected in part based on the use of 70% as the threshold for implementation-readiness set by Gavi as part of eligibility for Gavi support.

Supplementary Equation 1. **Year of Introduction Total Score Sample Calculation.** Total scores were calculated based on a 20% weighting of the RHD disease burden score and 40% weighting of each of the vaccine adoption history score and vaccine delivery infrastructure score, each normalized to its respective maximum score (see Table S-1 and Table S-2 above). The "Max Years to Introduction" (maximum number of years after first market launch for a country to adopt the vaccine in its NIP) differs for the base case, conservative and optimistic timeline scenarios (15, 20 and 8 years, respectively).

$$Year\ of\ Introduction\ Total\ Score = [((BURDEN\ SCORE)/(Maximum\ Burden\ Score) \times 20\%) + ((ADTOPTION\ SCORE)/(Maximum\ Adoption\ Score) \times 40\%) + ((INFRASTRUCTURE\ SCORE)/(Maximum\ Infrastructure\ Score) \times 40\%)] \times\ Max\ Years\ to\ Introduction$$

Supplementary Table 2. Year of Introduction Scoring Results. Year of introduction analysis scores were calculated by adding the integer value assigned to each country according to the criteria outlined in Table S-1.

	RHD Disease Burden		Vaccine Adoption History (Hib, Rota and PCV)							Vaccine Delivery Infrastructure	
**Country**	Age Standardized RHD Incidence (new cases per 100,000)	BURDEN SCORE	2019 Hib3 Coverage	Hib3 Coverage Score	2019 RotaC Coverage	RotaC Coverage Score	2019 PCV3 Coverage	PCV3 Coverage Score	ADOPTION HISTORY SCORE	2019 DTP3 Coverage	INFRA-STRUCTURE SCORE
Afghanistan	22.01836	2	66	0	60	0	65	0	0	66	4
Albania	21.72603	2	99	0	Planned intro in 2019	2	98	0	2	99	0
Algeria	17.98196	2	91	0	Not in NIP	4	91	0	4	91	0
Angola	29.06774	0	59	0	65	0	67	0	0	57	4
Antigua and Barbuda	21.29513	2	95	0	Not in NIP	4	Given in special circumstances	2	6	95	0
Argentina	22.34303	2	86	0	80	0	88	0	0	86	2
Armenia	24.54919	0	92	0	93	0	92	0	0	92	0
Australia	1.80753	4	94	0	87	0	95	0	0	95	0
Austria	2.21665	4	85	0	61	0	0	2	2	85	2
Azerbaijan	21.89797	2	95	0	Not in NIP	4	95	0	4	94	0
Bahamas, The	21.97230	2	90	0	70	0	90	0	0	86	2
Bahrain	1.43917	4	99	0	98	0	98	0	0	99	0
Bangladesh	19.44290	2	98	0	Not in NIP	4	97	0	4	98	0
Barbados	21.60814	2	95	0	Not in NIP	4	89	0	4	90	0
Belarus	3.11238	4	9	0	Not in NIP	4	Given in special circumstances	2	6	98	0
Belgium	2.16468	4	97	0	87	0	94	0	0	98	0
Belize	21.28747	2	96	0	Not in NIP	4	Not in NIP	4	8	98	0
Benin	25.57000	0	76	0	Not in NIP	4	73	0	4	76	2
Bhutan	18.79945	2	97	0	Not in NIP	4	Not in NIP	4	8	97	0
Bolivia	21.34830	2	83	0	87	0	83	0	0	75	2
Bosnia and Herzegovina	2.70813	4	62	0	Not in NIP	4	Given in special circumstances	2	6	73	2
Botswana	28.85023	0	95	0	87	0	91	0	0	95	0

	RHD Disease Burden		Vaccine Adoption History (Hib, Rota and PCV)							Vaccine Delivery Infrastructure	
Country	Age Standardized RHD Incidence (new cases per 100,000)	BURDEN SCORE	2019 Hib3 Coverage	Hib3 Coverage Score	2019 RotaC Coverage	RotaC Coverage Score	2019 PCV3 Coverage	PCV3 Coverage Score	ADOPTION HISTORY SCORE	2019 DTP3 Coverage	INFRA-STRUCTURE SCORE
Brazil	21.77207	2	83	0	80	0	84	0	0	73	2
Brunei Darussalam	2.49380	4	99	0	Not in NIP	4	Given in special circumstances	2	6	99	0
Bulgaria	2.64634	4	92	0	31	0	88	0	0	92	0
Burkina Faso	26.08265	0	91	0	91	0	91	0	0	91	0
Burundi	34.05402	0	90	0	92	0	90	0	0	93	0
Cabo Verde	25.67789	0	99	0	Not in NIP	4	Not in NIP	4	8	96	0
Cambodia	22.23125	2	92	0	Not in NIP	4	84	0	4	92	0
Cameroon	25.63998	0	79	0	78	0	79	0	0	67	4
Canada	1.94298	4	91	0	79	0	81	0	0	91	0
Central African Republic	30.47032	0	47	0	Planned intro in 2020	2	73	0	2	47	4
Chad	25.49099	0	41	0	Not in NIP	4	Not in NIP	4	8	50	4
Chile	2.77138	4	95	0	Not in NIP	4	93	0	4	96	0
China	17.46686	2	Not in NIP	4	Not in NIP	4	Not in NIP	4	12	99	0
Colombia	1.44221	4	92	0	90	0	94	0	0	92	0
Comoros	32.74771	0	91	0	Not in NIP	4	Planned intro in 2021	2	6	91	0
Congo, Dem. Rep.	29.44865	0	81	0	Planned intro in 2019	2	94	0	2	57	4
Congo, Rep.	29.81299	0	75	0	72	0	83	0	0	79	2
Costa Rica	22.46202	2	94	0	Planned intro in 2019	2	96	0	2	95	0
Côte d'Ivoire	26.93673	0	82	0	59	0	94	0	0	84	2
Croatia	1.95441	4	94	0	Not in NIP	4	Not in NIP	4	8	94	0
Cuba	21.65631	2	99	0	Not in NIP	4	Not in NIP	4	8	99	0
Cyprus	2.46843	4	97	0	Not in NIP	4	81	0	4	96	0
Czech Republic / Czechia	2.54049	4	94	0	Not in NIP	4	Not in NIP	4	8	97	0

	RHD Disease Burden		Vaccine Adoption History (Hib, Rota and PCV)							Vaccine Delivery Infrastructure	
**Country**	Age Standardized RHD Incidence (new cases per 100,000)	BURDEN SCORE	2019 Hib3 Coverage	Hib3 Coverage Score	2019 RotaC Coverage	RotaC Coverage Score	2019 PCV3 Coverage	PCV3 Coverage Score	ADOPTION HISTORY SCORE	2019 DTP3 Coverage	INFRA-STRUCTURE SCORE
Denmark	1.46382	4	97	0	Not in NIP	4	96	0	4	97	0
Djibouti	31.51769	0	84	0	87	0	84	0	0	85	2
Dominican Republic	21.19340	2	90	0	82	0	70	0	0	89	2
Ecuador	20.78718	2	85	0	85	0	85	0	0	85	2
Egypt, Arab Rep.	19.97120	2	95	0	Not in NIP	4	Not in NIP	4	8	95	0
El Salvador	21.94049	2	81	0	82	0	75	0	0	81	2
Equatorial Guinea	28.35932	0	25	0	Not in NIP	4	Not in NIP	4	8	53	4
Eritrea	33.21859	0	95	0	96	0	95	0	0	95	0
Estonia	3.17136	4	92	0	85	0	92	0	0	91	0
Eswatini	28.53253	0	90	0	90	0	88	0	0	90	0
Ethiopia	31.52785	0	72	0	79	0	67	0	0	69	4
Fiji	25.60089	0	99	0	99	0	99	0	0	99	0
Finland	1.04441	4	91	0	82	0	88	0	0	91	0
France	1.91610	4	95	0	Not in NIP	4	92	0	4	96	0
Gabon	29.36547	0	70	0	Not in NIP	4	Not in NIP	4	8	70	2
Gambia, The	27.47223	0	93	0	93	0	93	0	0	88	2
Georgia	22.55282	2	93	0	79	0	81	0	0	94	0
Germany	2.45338	4	92	0	68	0	84	0	0	93	0
Ghana	25.79695	0	97	0	94	0	96	0	0	97	0
Greece	1.51671	4	99	0	20	0	96	0	0	99	0
Grenada	21.90839	2	96	0	Not in NIP	4	Not in NIP	4	8	92	0
Guatemala	21.36208	2	86	0	87	0	85	0	0	85	2
Guinea	25.66104	0	45	0	Not in NIP	4	Not in NIP	4	8	47	4
Guinea-Bissau	27.44295	0	88	0	88	0	88	0	0	84	2
Guyana	22.71666	2	95	0	91	0	91	0	0	99	0
Haiti	23.20035	2	64	0	58	0	1	0	0	51	4

	RHD Disease Burden		Vaccine Adoption History (Hib, Rota and PCV)							Vaccine Delivery Infrastructure	
**Country**	Age Standardized RHD Incidence (new cases per 100,000)	BURDEN SCORE	2019 Hib3 Coverage	Hib3 Coverage Score	2019 RotaC Coverage	RotaC Coverage Score	2019 PCV3 Coverage	PCV3 Coverage Score	ADOPTION HISTORY SCORE	2019 DTP3 Coverage	INFRA-STRUCTURE SCORE
Honduras	22.37613	2	90	0	91	0	90	0	0	87	2
Hungary	2.88892	4	99	0	Not in NIP	4	99	0	4	99	0
Iceland	1.55307	4	91	0	Not in NIP	4	90	0	4	91	0
India	19.19006	2	89	0	35	0	6	0	0	91	0
Indonesia	5.98367	4	79	0	Not in NIP	4	Region-specific	2	6	85	2
Iran, Islamic Rep.	17.49707	2	99	0	Not in NIP	4	Given in special circumstances	2	6	99	0
Iraq	21.21683	2	84	0	60	0	32	0	0	84	2
Ireland	1.53373	4	94	0	89	0	90	0	0	94	0
Israel	1.95476	4	98	0	81	0	94	0	0	98	0
Italy	2.16537	4	94	0	19	0	92	0	0	95	0
Jamaica	21.07132	2	98	0	Not in NIP	4	Given in special circumstances	2	6	96	0
Japan	1.28272	4	99	0	Not in NIP	4	98	0	4	98	0
Jordan	1.01442	4	96	0	93	0	Not in NIP	4	4	89	2
Kazakhstan	3.20263	4	98	0	Not in NIP	4	95	0	4	97	0
Kenya	31.15829	0	92	0	78	0	81	0	0	92	0
Kiribati	28.94964	0	95	0	97	0	94	0	0	97	0
Korea, Dem. People's Rep.	22.68874	2	97	0	Not in NIP	4	Not in NIP	4	8	97	0
Korea, Rep.	1.13672	4	98	0	Not in NIP	4	97	0	4	98	0
Kuwait	1.10125	4	99	0	3	0	99	0	0	91	0
Kyrgyz Republic	22.88559	2	92	0	Planned intro in 2019	2	92	0	2	95	0
Lao PDR	24.41440	0	68	0	Planned intro in 2020	2	83	0	2	68	4
Latvia	2.86703	4	96	0	79	0	82	0	0	99	0
Lebanon	1.55585	4	85	0	Not in NIP	4	82	0	4	83	2
Lesotho	29.08751	0	93	0	70	0	93	0	0	87	2
Liberia	27.62787	0	84	0	74	0	84	0	0	74	2
Libya	20.53733	2	97	0	97	0	96	0	0	73	2

Country	RHD Disease Burden		Vaccine Adoption History (Hib, Rota and PCV)							Vaccine Delivery Infrastructure	
	Age Standardized RHD Incidence (new cases per 100,000)	BURDEN SCORE	2019 Hib3 Coverage	Hib3 Coverage Score	2019 RotaC Coverage	RotaC Coverage Score	2019 PCV3 Coverage	PCV3 Coverage Score	ADOPTION HISTORY SCORE	2019 DTP3 Coverage	INFRA-STRUCTURE SCORE
Lithuania	2.86404	4	92	0	1	0	82	0	0	92	0
Luxembourg	2.25174	4	99	0	89	0	96	0	0	99	0
Madagascar	33.26001	0	75	0	78	0	75	0	0	79	2
Malawi	35.89247	0	92	0	90	0	92	0	0	95	0
Malaysia	23.47665	2	99	0	Not in NIP	4	Not in NIP	4	8	98	0
Maldives	21.05804	2	99	0	Not in NIP	4	Not in NIP	4	8	99	0
Mali	26.18946	0	71	0	55	0	68	0	0	77	2
Malta	1.80304	4	97	0	Planned intro in 2019	2	Not in NIP	4	6	98	0
Mauritania	25.40416	0	81	0	76	0	77	0	0	81	2
Mauritius	21.85060	2	97	0	95	0	96	0	0	96	0
Mexico	8.98218	4	88	0	77	0	88	0	0	82	2
Micronesia, Fed. Sts.	28.64251	0	59	0	52	0	67	0	0	78	2
Moldova	2.98680	4	92	0	75	0	94	0	0	91	0
Mongolia	24.15871	2	99	0	Not in NIP	4	26	0	4	98	0
Montenegro	2.49229	4	87	0	Not in NIP	4	Not in NIP	4	8	86	2
Morocco	21.33196	2	99	0	99	0	99	0	0	99	0
Mozambique	36.10258	0	80	0	80	0	80	0	0	88	2
Myanmar	24.18544	2	91	0	Not in NIP	4	91	0	4	90	0
Namibia	27.66534	0	89	0	92	0	61	0	0	87	2
Nepal	20.46001	2	91	0	Not in NIP	4	82	0	4	93	0
Netherlands	1.82670	4	93	0	Not in NIP	4	93	0	4	94	0
New Zealand	2.47518	4	92	0	86	0	96	0	0	92	0
Nicaragua	21.99224	2	98	0	98	0	98	0	0	98	0
Niger	25.76499	0	89	0	89	0	79	0	0	81	2
Nigeria	25.23289	0	57	0	Planned intro in 2019	2	57	0	2	57	4
North Macedonia	2.19387	4	91	0	Not in NIP	4	Not in NIP	4	8	92	0

	RHD Disease Burden		Vaccine Adoption History (Hib, Rota and PCV)							Vaccine Delivery Infrastructure	
Country	Age Standardized RHD Incidence (new cases per 100,000)	BURDEN SCORE	2019 Hib3 Coverage	Hib3 Coverage Score	2019 RotaC Coverage	RotaC Coverage Score	2019 PCV3 Coverage	PCV3 Coverage Score	ADOPTION HISTORY SCORE	2019 DTP3 Coverage	INFRA-STRUCTURE SCORE
Norway	1.01772	4	96	0	93	0	94	0	0	97	0
Oman	1.31332	4	99	0	Not in NIP	4	99	0	4	99	0
Pakistan	21.21368	2	75	0	58	0	79	0	0	75	2
Panama	20.80452	2	88	0	95	0	92	0	0	88	2
Papua New Guinea	29.01053	0	61	0	Not in NIP	4	43	0	4	35	4
Paraguay	22.03975	2	88	0	91	0	94	0	0	86	2
Peru	20.83701	2	84	0	85	0	82	0	0	88	2
Philippines	19.37087	2	65	0	Not in NIP	4	Not in entire country (60% coverage)	2	6	65	4
Poland	3.04748	4	95	0	Not in NIP	4	60	0	4	95	0
Portugal	1.87051	4	99	0	Not in NIP	4	98	0	4	99	0
Qatar	1.30209	4	98	0	95	0	98	0	0	98	0
Romania	3.00444	4	86	0	Not in NIP	4	0	2	6	88	2
Russian Federation	3.52581	4	Given in special circumstance s	2	Not in NIP	4	82	0	6	97	0
Rwanda	32.06587	0	97	0	98	0	97	0	0	98	0
Samoa	27.66043	0	34	0	Not in NIP	4	Not in NIP	4	8	58	4
São Tomé and Principe	27.63366	0	95	0	95	0	95	0	0	95	0
Saudi Arabia	1.28493	4	96	0	97	0	98	0	0	96	0
Senegal	26.84276	0	82	0	80	0	81	0	0	93	0
Serbia	2.30409	4	96	0	Not in NIP	4	48	0	4	97	0
Seychelles	21.72450	2	99	0	99	0	16	0	0	99	0
Sierra Leone	27.58495	0	90	0	92	0	90	0	0	95	0
Singapore	1.00773	4	96	0	Not in NIP	4	82	0	4	96	0
Slovak Republic / Slovakia	2.75259	4	96	0	Not in NIP	4	96	0	4	97	0
Slovenia	2.58996	4	93	0	Not in NIP	4	60	0	4	95	0

	RHD Disease Burden		Vaccine Adoption History (Hib, Rota and PCV)							Vaccine Delivery Infrastructure	
**Country**	Age Standardized RHD Incidence (new cases per 100,000)	BURDEN SCORE	2019 Hib3 Coverage	Hib3 Coverage Score	2019 RotaC Coverage	RotaC Coverage Score	2019 PCV3 Coverage	PCV3 Coverage Score	ADOPTION HISTORY SCORE	2019 DTP3 Coverage	INFRA-STRUCTURE SCORE
Solomon Islands	28.65724	0	85	0	Not in NIP	4	84	0	4	94	0
Somalia	33.70852	0	42	0	Not in NIP	4	Not in NIP	4	8	42	4
South Africa	27.66179	0	74	0	70	0	73	0	0	77	2
South Sudan	31.52766	0	49	0	Not in NIP	4	Not in NIP	4	8	49	4
Spain	2.28191	4	94	0	Not in NIP	4	93	0	4	96	0
Sri Lanka	1.84733	4	99	0	Not in NIP	4	Not in NIP	4	8	99	0
St. Lucia	22.19574	2	95	0	Not in NIP	4	Given in special circumstances	2	6	92	0
St. Vincent and the Grenadines	21.64683	2	97	0	Not in NIP	4	Not in NIP	4	8	97	0
Sudan	20.77935	2	93	0	94	0	93	0	0	93	0
Suriname	20.98622	2	95	0	Not in NIP	4	Not in NIP	4	8	77	2
Sweden	1.41812	4	97	0	Not in NIP	4	97	0	4	98	0
Switzerland	1.67222	4	95	0	Not in NIP	4	85	0	4	96	0
Syrian Arab Republic	19.54711	2	48	0	Not in NIP	4	Not in NIP	4	8	54	4
Tajikistan	23.17498	2	96	0	96	0	Not in NIP	4	4	97	0
Tanzania	33.04759	0	98	0	98	0	98	0	0	89	2
Thailand	21.09732	2	Not in NIP	4	Not in NIP	4	Not in NIP	4	12	97	0
Timor-Leste	23.71845	2	83	0	Planned intro in 2019	2	Not in NIP	4	6	83	2
Togo	27.11331	0	88	0	89	0	88	0	0	84	2
Tonga	31.68581	0	81	0	Planned intro in 2019	2	Planned intro in 2019	2	4	99	0
Trinidad and Tobago	22.32510	2	99	0	Not in NIP	4	99	0	4	93	0
Tunisia	1.44863	4	97	0	Not in NIP	4	Not in NIP	4	8	92	0
Turkey	1.10588	4	98	0	Not in NIP	4	97	0	4	99	0
Turkmenistan	22.28020	2	99	0	Planned intro in 2019	2	Planned intro in 2019	2	4	99	0
Uganda	32.53248	0	93	0	36	0	92	0	0	93	0

	RHD Disease Burden		Vaccine Adoption History (Hib, Rota and PCV)							Vaccine Delivery Infrastructure	
**Country**	Age Standardized RHD Incidence (new cases per 100,000)	BURDEN SCORE	2019 Hib3 Coverage	Hib3 Coverage Score	2019 RotaC Coverage	RotaC Coverage Score	2019 PCV3 Coverage	PCV3 Coverage Score	ADOPTION HISTORY SCORE	2019 DTP3 Coverage	INFRA-STRUCTURE SCORE
Ukraine	2.65899	4	39	0	Not in NIP	4	Not in NIP	4	8	80	2
United Arab Emirates	19.90254	2	99	0	99	0	99	0	0	99	0
United Kingdom	1.43889	4	94	0	91	0	92	0	0	93	0
United States	2.27463	4	92	0	73	0	92	0	0	94	0
Uruguay	3.66885	4	91	0	Not in NIP	4	93	0	4	94	0
Uzbekistan	23.05046	2	98	0	84	0	96	0	0	96	0
Vanuatu	31.11954	0	85	0	Not in NIP	4	Not in NIP	4	8	90	0
Venezuela, RB	1.46080	4	60	0	0	2	0	2	4	64	4
Vietnam	2.50439	4	75	0	Not in NIP	4	Not in NIP	4	8	89	2
Yemen, Rep.	21.98410	2	65	0	64	0	64	0	0	73	2
Zambia	31.98653	0	90	0	91	0	90	0	0	88	2
Zimbabwe	28.07637	0	89	0	90	0	89	0	0	90	0

Supplementary Table 3. Year of Introduction Total Scores for the Base Case, Conservative and Optimistic Timeline Scenarios. The equation used to calculate Year of Introduction Total Score is shown above in Equation S-1. Countries with a score of “0” were assigned to Group 1 (integer value of 0) for all 3 dimensions (see Table S-1).

Country	Year of Introduction Total Scores		
	Base Case	Conservative	Optimistic
Afghanistan	8	10	4
Albania	3	3	1
Algeria	4	5	2
Angola	6	8	3
Antigua and Barbuda	5	6	2
Argentina	5	6	2
Armenia	0	0	0
Australia	3	4	2
Austria	7	9	4
Azerbaijan	4	5	2
Bahamas, The	5	6	2
Bahrain	3	4	2
Bangladesh	4	5	2
Barbados	4	5	2
Belarus	6	8	3
Belgium	3	4	2
Belize	6	7	3
Benin	5	7	3
Bhutan	6	7	3
Bolivia	5	6	2
Bosnia and Herzegovina	9	12	5
Botswana	0	0	0
Brazil	5	6	2
Brunei Darussalam	6	8	3
Bulgaria	3	4	2
Burkina Faso	0	0	0
Burundi	0	0	0
Cabo Verde	4	5	2
Cambodia	4	5	2
Cameroon	6	8	3
Canada	3	4	2
Central African Republic	7	9	4
Chad	10	13	5
Chile	5	7	3
China	8	10	4
Colombia	3	4	2
Comoros	3	4	2
Congo, Dem. Rep.	7	9	4
Congo, Rep.	3	4	2

Country	Year of Introduction Total Scores		
	Base Case	Conservative	Optimistic
Costa Rica	3	3	1
Côte d'Ivoire	3	4	2
Croatia	7	9	4
Cuba	6	7	3
Cyprus	5	7	3
Czech Republic / Czechia	7	9	4
Denmark	5	7	3
Djibouti	3	4	2
Dominican Republic	5	6	2
Ecuador	5	6	2
Egypt, Arab Rep.	6	7	3
El Salvador	5	6	2
Equatorial Guinea	10	13	5
Eritrea	0	0	0
Estonia	3	4	2
Eswatini	0	0	0
Ethiopia	6	8	3
Fiji	0	0	0
Finland	3	4	2
France	5	7	3
Gabon	7	9	4
Gambia, The	3	4	2
Georgia	2	2	1
Germany	3	4	2
Ghana	0	0	0
Greece	3	4	2
Grenada	6	7	3
Guatemala	5	6	2
Guinea	10	13	5
Guinea-Bissau	3	4	2
Guyana	2	2	1
Haiti	8	10	4
Honduras	5	6	2
Hungary	5	7	3
Iceland	5	7	3
India	2	2	1
Indonesia	9	12	5
Iran, Islamic Rep.	5	6	2
Iraq	5	6	2
Ireland	3	4	2
Israel	3	4	2
Italy	3	4	2
Jamaica	5	6	2
Japan	5	7	3

Country	Year of Introduction Total Scores		
	Base Case	Conservative	Optimistic
Jordan	8	11	4
Kazakhstan	5	7	3
Kenya	0	0	0
Kiribati	0	0	0
Korea, Dem. People’s Rep.	6	7	3
Korea, Rep.	5	7	3
Kuwait	3	4	2
Kyrgyz Republic	3	3	1
Lao PDR	7	9	4
Latvia	3	4	2
Lebanon	8	11	4
Lesotho	3	4	2
Liberia	3	4	2
Libya	5	6	2
Lithuania	3	4	2
Luxembourg	3	4	2
Madagascar	3	4	2
Malawi	0	0	0
Malaysia	6	7	3
Maldives	6	7	3
Mali	3	4	2
Malta	6	8	3
Mauritania	3	4	2
Mauritius	2	2	1
Mexico	6	8	3
Micronesia, Fed. Sts.	3	4	2
Moldova	3	4	2
Mongolia	4	5	2
Montenegro	10	13	5
Morocco	2	2	1
Mozambique	3	4	2
Myanmar	4	5	2
Namibia	3	4	2
Nepal	4	5	2
Netherlands	5	7	3
New Zealand	3	4	2
Nicaragua	2	2	1
Niger	3	4	2
Nigeria	7	9	4
North Macedonia	7	9	4
Norway	3	4	2
Oman	5	7	3
Pakistan	5	6	2
Panama	5	6	2
Papua New Guinea	8	11	4

Country	Year of Introduction Total Scores		
	Base Case	Conservative	Optimistic
Paraguay	5	6	2
Peru	5	6	2
Philippines	11	14	6
Poland	5	7	3
Portugal	5	7	3
Qatar	3	4	2
Romania	9	12	5
Russian Federation	6	8	3
Rwanda	0	0	0
Samoa	10	13	5
São Tomé and Principe	0	0	0
Saudi Arabia	3	4	2
Senegal	0	0	0
Serbia	5	7	3
Seychelles	2	2	1
Sierra Leone	0	0	0
Singapore	5	7	3
Slovak Republic / Slovakia	5	7	3
Slovenia	5	7	3
Solomon Islands	2	3	1
Somalia	10	13	5
South Africa	3	4	2
South Sudan	10	13	5
Spain	5	7	3
Sri Lanka	7	9	4
St. Lucia	5	6	2
St. Vincent and the Grenadines	6	7	3
Sudan	2	2	1
Suriname	9	11	5
Sweden	5	7	3
Switzerland	5	7	3
Syrian Arab Republic	12	15	6
Tajikistan	4	5	2
Tanzania	3	4	2
Thailand	8	10	4
Timor-Leste	8	10	4
Togo	3	4	2
Tonga	2	3	1
Trinidad and Tobago	4	5	2
Tunisia	7	9	4
Turkey	5	7	3
Turkmenistan	4	5	2
Uganda	0	0	0
Ukraine	10	13	5

Country	Year of Introduction Total Scores		
	Base Case	Conservative	Optimistic
United Arab Emirates	2	2	1
United Kingdom	3	4	2
United States	3	4	2
Uruguay	5	7	3
Uzbekistan	2	2	1
Vanuatu	4	5	2
Venezuela, RB	11	15	6
Vietnam	10	13	5
Yemen, Rep.	5	6	2
Zambia	3	4	2
Zimbabwe	0	0	0

Supplementary Table 4. Manual Adjustment (In Number of Years) For Each of the Vaccine Developer Scenarios. Manual adjustments to the Year of Introduction Total Score (Table S-2) are based on interviews with biopharmaceutical industry experts and vaccine decisionmakers in a subset of countries. A positive value represents a delay in the year of introduction as calculated using the scoring system (Table S-1) while a negative value represents an acceleration of the year of introduction as calculated using the scoring system. Rationale for manual adjustments is provided in the Methods section of the manuscript.

Country	Manual Adjustment (number of years)		
	Pharma MNC Global Rollout	Pharma MNC Staged Rollout	DCVM Staged Rollout
Afghanistan	2	2	2
Albania	0	0	0
Algeria	0	0	0
Angola	0	0	0
Antigua and Barbuda	0	0	0
Argentina	0	0	0
Armenia	0	0	0
Australia	-2	-2	-2
Austria	0	2	0
Azerbaijan	0	0	0
Bahamas, The	0	0	0
Bahrain	0	0	0
Bangladesh	1	1	1
Barbados	0	0	0
Belarus	0	0	0
Belgium	0	2	0
Belize	0	0	0
Benin	2	2	2
Bhutan	0	0	0
Bolivia	0	0	0

Country	Manual Adjustment (number of years)		
	Pharma MNC Global Rollout	Pharma MNC Staged Rollout	DCVM Staged Rollout
Bosnia and Herzegovina	0	0	0
Botswana	0	0	0
Brazil	0	0	0
Brunei Darussalam	0	0	0
Bulgaria	0	2	0
Burkina Faso	2	2	2
Burundi	2	2	2
Cabo Verde	0	0	0
Cambodia	2	2	2
Cameroon	2	2	2
Canada	-2	2	-2
Central African Republic	2	2	2
Chad	2	2	2
Chile	0	0	0
China	-2	-2	-2
Colombia	0	0	0
Comoros	2	2	2
Congo, Dem. Rep.	2	2	2
Congo, Rep.	2	2	2
Costa Rica	0	0	0
Côte d'Ivoire	2	2	2
Croatia	0	2	0
Cuba	0	0	0
Cyprus	0	2	0
Czech Republic / Czechia	0	2	0
Denmark	0	2	0
Djibouti	2	2	2
Dominican Republic	0	0	0
Ecuador	0	0	0
Egypt, Arab Rep.	0	0	0
El Salvador	0	0	0
Equatorial Guinea	0	0	0
Eritrea	2	2	2
Estonia	0	2	0
Eswatini	0	0	0
Ethiopia	2	2	2
Fiji	0	0	0
Finland	0	2	0
France	-2	2	-2
Gabon	0	0	0
Gambia, The	2	2	2
Georgia	0	0	0

Country	Manual Adjustment (number of years)		
	Pharma MNC Global Rollout	Pharma MNC Staged Rollout	DCVM Staged Rollout
Germany	-2	2	-2
Ghana	2	2	2
Greece	0	2	0
Grenada	0	0	0
Guatemala	0	0	0
Guinea	2	2	2
Guinea-Bissau	2	2	2
Guyana	0	0	0
Haiti	1	1	1
Honduras	0	0	0
Hungary	0	2	0
Iceland	0	0	0
India	0	0	0
Indonesia	0	0	0
Iran, Islamic Rep.	0	0	0
Iraq	0	0	0
Ireland	0	2	0
Israel	0	0	0
Italy	-2	2	-2
Jamaica	0	0	0
Japan	0	0	0
Jordan	0	0	0
Kazakhstan	0	0	0
Kenya	2	2	2
Kiribati	0	0	0
Korea, Dem. People's Rep.	2	2	2
Korea, Rep.	-1	-1	-1
Kuwait	0	0	0
Kyrgyz Republic	2	2	2
Lao PDR	0	0	0
Latvia	0	2	0
Lebanon	0	0	0
Lesotho	2	2	2
Liberia	2	2	2
Libya	0	0	0
Lithuania	0	2	0
Luxembourg	0	2	0
Madagascar	2	2	2
Malawi	2	2	2
Malaysia	0	0	0
Maldives	0	0	0
Mali	2	2	2
Malta	0	2	0
Mauritania	2	2	2

Country	Manual Adjustment (number of years)		
	Pharma MNC Global Rollout	Pharma MNC Staged Rollout	DCVM Staged Rollout
Mauritius	0	0	0
Mexico	0	0	0
Micronesia, Fed. Sts.	0	0	0
Moldova	0	0	0
Mongolia	0	0	0
Montenegro	0	0	0
Morocco	0	0	0
Mozambique	2	2	2
Myanmar	2	2	2
Namibia	0	0	0
Nepal	2	2	2
Netherlands	0	2	0
New Zealand	-2	-2	-2
Nicaragua	0	0	0
Niger	2	2	2
Nigeria	0	0	0
North Macedonia	0	0	0
Norway	0	0	0
Oman	0	0	0
Pakistan	2	2	2
Panama	0	0	0
Papua New Guinea	-2	-2	-2
Paraguay	0	0	0
Peru	0	0	0
Philippines	0	0	0
Poland	0	2	0
Portugal	0	2	0
Qatar	0	0	0
Romania	0	2	0
Russian Federation	0	0	0
Rwanda	2	2	2
Samoa	0	0	0
São Tomé and Principe	0	0	0
Saudi Arabia	0	0	0
Senegal	2	2	2
Serbia	0	0	0
Seychelles	0	0	0
Sierra Leone	2	2	2
Singapore	0	0	0
Slovak Republic / Slovakia	0	2	0
Slovenia	0	2	0
Solomon Islands	0	0	0
Somalia	2	2	2
South Africa	-1	-1	-1

Country	Manual Adjustment (number of years)		
	Pharma MNC Global Rollout	Pharma MNC Staged Rollout	DCVM Staged Rollout
South Sudan	2	2	2
Spain	-2	2	-2
Sri Lanka	0	0	0
St. Lucia	0	0	0
St. Vincent and the Grenadines	0	0	0
Sudan	2	2	2
Suriname	0	0	0
Sweden	0	2	0
Switzerland	0	0	0
Syrian Arab Republic	2	2	2
Tajikistan	2	2	2
Tanzania	2	2	2
Thailand	0	0	0
Timor-Leste	0	0	0
Togo	2	2	2
Tonga	0	0	0
Trinidad and Tobago	0	0	0
Tunisia	0	0	0
Turkey	0	0	0
Turkmenistan	0	0	0
Uganda	2	2	2
Ukraine	0	0	0
United Arab Emirates	0	0	0
United Kingdom	-3	2	-3
United States	-3	4	-3
Uruguay	0	0	0
Uzbekistan	0	0	0
Vanuatu	0	0	0
Venezuela, RB	0	0	0
Vietnam	0	0	0
Yemen, Rep.	2	2	2
Zambia	2	2	2
Zimbabwe	2	2	2

Supplementary Table 5. Income Group Level (or Gavi-Eligibility) and Year of Introduction for Each Country in the Pharma MNC Global Rollout, Pharma MNC Staged Rollout and DCVM Staged Rollout Scenarios. Year of Introduction was calculated based on adding the Year of Introduction Total Score (Equation S-1 and Table S-2) with the Manual Adjustment (Table S-3). In the **Pharma MNC** Staged Rollout scenario first market entry is 2033. In staged rollouts by **Pharma MNC** and DCVMs, first market entry is in 2033 for all countries except high-income countries (HICs) and 2038 for HIC markets.

Country	Income Group/Gavi-eligibility	Year of Introduction		
		Pharma MNC Global Rollout	Pharma MNC Staged Rollout	DCVM Staged Rollout
Afghanistan	Gavi	2043	2043	2043
Albania	Upper middle income	2036	2036	2036
Algeria	Upper middle income	2037	2037	2037
Angola	Lower middle income	2039	2039	2039
Antigua and Barbuda	High income	2038	2043	2043
Argentina	Upper middle income	2038	2038	2038
Armenia	Upper middle income	2033	2033	2033
Australia	High income	2034	2039	2039
Austria	High income	2040	2045	2047
Azerbaijan	Upper middle income	2037	2037	2037
Bahamas, The	High income	2038	2043	2043
Bahrain	High income	2036	2041	2041
Bangladesh	Gavi	2038	2038	2038
Barbados	High income	2037	2042	2042
Belarus	Upper middle income	2039	2039	2039
Belgium	High income	2036	2041	2043
Belize	Upper middle income	2039	2039	2039
Benin	Gavi	2040	2040	2040
Bhutan	Lower middle income	2039	2039	2039
Bolivia	Lower middle income	2038	2038	2038
Bosnia and Herzegovina	Upper middle income	2042	2042	2042
Botswana	Upper middle income	2033	2033	2033
Brazil	Upper middle income	2038	2038	2038
Brunei Darussalam	High income	2039	2044	2044
Bulgaria	Upper middle income	2036	2036	2038
Burkina Faso	Gavi	2035	2035	2035
Burundi	Gavi	2035	2035	2035
Cabo Verde	Lower middle income	2037	2037	2037
Cambodia	Gavi	2039	2039	2039
Cameroon	Gavi	2041	2041	2041
Canada	High income	2034	2039	2043
Central African Republic	Gavi	2042	2042	2042
Chad	Gavi	2045	2045	2045
Chile	High income	2038	2043	2043

Country	Income Group/Gavi-eligibility	Year of Introduction		
		Pharma MNC Global Rollout	Pharma MNC Staged Rollout	DCVM Staged Rollout
China	Upper middle income	2039	2039	2039
Colombia	Upper middle income	2036	2036	2036
Comoros	Gavi	2038	2038	2038
Congo, Dem. Rep.	Gavi	2042	2042	2042
Congo, Rep.	Gavi	2038	2038	2038
Costa Rica	Upper middle income	2036	2036	2036
Côte d'Ivoire	Gavi	2038	2038	2038
Croatia	High income	2040	2045	2047
Cuba	Upper middle income	2039	2039	2039
Cyprus	High income	2038	2043	2045
Czech Republic / Czechia	High income	2040	2045	2047
Denmark	High income	2038	2043	2045
Djibouti	Gavi	2038	2038	2038
Dominican Republic	Upper middle income	2038	2038	2038
Ecuador	Upper middle income	2038	2038	2038
Egypt, Arab Rep.	Lower middle income	2039	2039	2039
El Salvador	Lower middle income	2038	2038	2038
Equatorial Guinea	Upper middle income	2043	2043	2043
Eritrea	Gavi	2035	2035	2035
Estonia	High income	2036	2041	2043
Eswatini	Lower middle income	2033	2033	2033
Ethiopia	Gavi	2041	2041	2041
Fiji	Upper middle income	2033	2033	2033
Finland	High income	2036	2041	2043
France	High income	2036	2041	2045
Gabon	Upper middle income	2040	2040	2040
Gambia, The	Gavi	2038	2038	2038
Georgia	Upper middle income	2035	2035	2035
Germany	High income	2034	2039	2043
Ghana	Gavi	2035	2035	2035
Greece	High income	2036	2041	2043
Grenada	Upper middle income	2039	2039	2039
Guatemala	Upper middle income	2038	2038	2038
Guinea	Gavi	2045	2045	2045
Guinea-Bissau	Gavi	2038	2038	2038
Guyana	Upper middle income	2035	2035	2035
Haiti	Gavi	2042	2042	2042
Honduras	Lower middle income	2038	2038	2038
Hungary	High income	2038	2043	2045
Iceland	High income	2038	2043	2043
India	Lower middle income	2035	2035	2035

Country	Income Group/Gavi-eligibility	Year of Introduction		
		Pharma MNC Global Rollout	Pharma MNC Staged Rollout	DCVM Staged Rollout
Indonesia	Lower middle income	2042	2042	2042
Iran, Islamic Rep.	Upper middle income	2038	2038	2038
Iraq	Upper middle income	2038	2038	2038
Ireland	High income	2036	2041	2043
Israel	High income	2036	2041	2041
Italy	High income	2034	2039	2043
Jamaica	Upper middle income	2038	2038	2038
Japan	High income	2038	2043	2043
Jordan	Upper middle income	2041	2041	2041
Kazakhstan	Upper middle income	2038	2038	2038
Kenya	Gavi	2035	2035	2035
Kiribati	Lower middle income	2033	2033	2033
Korea, Dem. People's Rep.	Gavi	2041	2041	2041
Korea, Rep.	High income	2037	2042	2042
Kuwait	High income	2036	2041	2041
Kyrgyz Republic	Gavi	2038	2038	2038
Lao PDR	Lower middle income	2040	2040	2040
Latvia	High income	2036	2041	2043
Lebanon	Upper middle income	2041	2041	2041
Lesotho	Gavi	2038	2038	2038
Liberia	Gavi	2038	2038	2038
Libya	Upper middle income	2038	2038	2038
Lithuania	High income	2036	2041	2043
Luxembourg	High income	2036	2041	2043
Madagascar	Gavi	2038	2038	2038
Malawi	Gavi	2035	2035	2035
Malaysia	Upper middle income	2039	2039	2039
Maldives	Upper middle income	2039	2039	2039
Mali	Gavi	2038	2038	2038
Malta	High income	2039	2044	2046
Mauritania	Gavi	2038	2038	2038
Mauritius	Upper middle income	2035	2035	2035
Mexico	Upper middle income	2039	2039	2039
Micronesia, Fed. Sts.	Lower middle income	2036	2036	2036
Moldova	Lower middle income	2036	2036	2036
Mongolia	Lower middle income	2037	2037	2037
Montenegro	Upper middle income	2043	2043	2043
Morocco	Lower middle income	2035	2035	2035
Mozambique	Gavi	2038	2038	2038
Myanmar	Gavi	2039	2039	2039
Namibia	Upper middle income	2036	2036	2036
Nepal	Gavi	2039	2039	2039

Country	Income Group/Gavi-eligibility	Year of Introduction		
		Pharma MNC Global Rollout	Pharma MNC Staged Rollout	DCVM Staged Rollout
Netherlands	High income	2038	2043	2045
New Zealand	High income	2034	2039	2039
Nicaragua	Lower middle income	2035	2035	2035
Niger	Gavi	2038	2038	2038
Nigeria	Lower middle income	2040	2040	2040
North Macedonia	Upper middle income	2040	2040	2040
Norway	High income	2036	2041	2041
Oman	High income	2038	2043	2043
Pakistan	Gavi	2040	2040	2040
Panama	High income	2038	2043	2043
Papua New Guinea	Lower middle income	2039	2039	2039
Paraguay	Upper middle income	2038	2038	2038
Peru	Upper middle income	2038	2038	2038
Philippines	Lower middle income	2044	2044	2044
Poland	High income	2038	2043	2045
Portugal	High income	2038	2043	2045
Qatar	High income	2036	2041	2041
Romania	Upper middle income	2042	2042	2044
Russian Federation	Upper middle income	2039	2039	2039
Rwanda	Gavi	2035	2035	2035
Samoa	Upper middle income	2043	2043	2043
São Tomé and Principe	Lower middle income	2033	2033	2033
Saudi Arabia	High income	2036	2041	2041
Senegal	Gavi	2035	2035	2035
Serbia	Upper middle income	2038	2038	2038
Seychelles	High income	2035	2040	2040
Sierra Leone	Gavi	2035	2035	2035
Singapore	High income	2038	2043	2043
Slovak Republic / Slovakia	High income	2038	2043	2045
Slovenia	High income	2038	2043	2045
Solomon Islands	Lower middle income	2035	2035	2035
Somalia	Gavi	2045	2045	2045
South Africa	Upper middle income	2035	2035	2035
South Sudan	Gavi	2045	2045	2045
Spain	High income	2036	2041	2045
Sri Lanka	Upper middle income	2040	2040	2040
St. Lucia	Upper middle income	2038	2038	2038
St. Vincent and the Grenadines	Upper middle income	2039	2039	2039
Sudan	Gavi	2037	2037	2037
Suriname	Upper middle income	2042	2042	2042
Sweden	High income	2038	2043	2045
Switzerland	High income	2038	2043	2043

Country	Income Group/Gavi-eligibility	Year of Introduction		
		Pharma MNC Global Rollout	Pharma MNC Staged Rollout	DCVM Staged Rollout
Syrian Arab Republic	Gavi	2047	2047	2047
Tajikistan	Gavi	2039	2039	2039
Tanzania	Gavi	2038	2038	2038
Thailand	Upper middle income	2041	2041	2041
Timor-Leste	Lower middle income	2041	2041	2041
Togo	Gavi	2038	2038	2038
Tonga	Upper middle income	2035	2035	2035
Trinidad and Tobago	High income	2037	2042	2042
Tunisia	Lower middle income	2040	2040	2040
Turkey	Upper middle income	2038	2038	2038
Turkmenistan	Upper middle income	2037	2037	2037
Uganda	Gavi	2035	2035	2035
Ukraine	Lower middle income	2043	2043	2043
United Arab Emirates	High income	2035	2040	2040
United Kingdom	High income	2033	2038	2043
United States	High income	2033	2038	2045
Uruguay	High income	2038	2043	2043
Uzbekistan	Lower middle income	2035	2035	2035
Vanuatu	Lower middle income	2037	2037	2037
Venezuela, RB	Upper middle income	2044	2044	2044
Vietnam	Lower middle income	2043	2043	2043
Yemen, Rep.	Gavi	2040	2040	2040
Zambia	Gavi	2038	2038	2038
Zimbabwe	Gavi	2035	2035	2035

Supplementary Table 6. Year of Introduction for Each Country in the Pharma MNC Global Rollout Scenario for each of the base case, conservative and optimistic timeline scenarios. Year of Introduction depends on the "Max Years to Introduction" (maximum number of years after first market launch for a country to adopt the vaccine in its NIP) which differs across the base case, conservative and optimistic timeline scenarios (15, 20 and 8 years, respectively), as outlined in Equation S-1.

	Year of Introduction for the Pharma MNC Global Rollout Scenario		
	Base Case Timeline Scenario	Conservative Timeline Scenario	Optimistic Timeline Scenario
Afghanistan	2043	2045	2036
Albania	2036	2036	2031
Algeria	2037	2038	2032
Angola	2039	2041	2033
Antigua and Barbuda	2038	2039	2032
Argentina	2038	2039	2032

	Year of Introduction for the Pharma MNC Global Rollout Scenario		
	Base Case Timeline Scenario	Conservative Timeline Scenario	Optimistic Timeline Scenario
Armenia	2033	2033	2030
Australia	2034	2035	2030
Austria	2040	2042	2034
Azerbaijan	2037	2038	2032
Bahamas, The	2038	2039	2032
Bahrain	2036	2037	2032
Bangladesh	2038	2039	2033
Barbados	2037	2038	2032
Belarus	2039	2041	2033
Belgium	2036	2037	2032
Belize	2039	2040	2033
Benin	2040	2042	2035
Bhutan	2039	2040	2033
Bolivia	2038	2039	2032
Bosnia and Herzegovina	2042	2045	2035
Botswana	2033	2033	2030
Brazil	2038	2039	2032
Brunei Darussalam	2039	2041	2033
Bulgaria	2036	2037	2032
Burkina Faso	2035	2035	2032
Burundi	2035	2035	2032
Cabo Verde	2037	2038	2032
Cambodia	2039	2040	2034
Cameroon	2041	2043	2035
Canada	2034	2035	2030
Central African Republic	2042	2044	2036
Chad	2045	2048	2037
Chile	2038	2040	2033
China	2039	2041	2032
Colombia	2036	2037	2032
Comoros	2038	2039	2034
Congo, Dem. Rep.	2042	2044	2036
Congo, Rep.	2038	2039	2034
Costa Rica	2036	2036	2031
Côte d'Ivoire	2038	2039	2034
Croatia	2040	2042	2034
Cuba	2039	2040	2033
Cyprus	2038	2040	2033
Czech Republic / Czechia	2040	2042	2034
Denmark	2038	2040	2033

	Year of Introduction for the Pharma MNC Global Rollout Scenario		
	Base Case Timeline Scenario	Conservative Timeline Scenario	Optimistic Timeline Scenario
Djibouti	2038	2039	2034
Dominican Republic	2038	2039	2032
Ecuador	2038	2039	2032
Egypt, Arab Rep.	2039	2040	2033
El Salvador	2038	2039	2032
Equatorial Guinea	2043	2046	2035
Eritrea	2035	2035	2032
Estonia	2036	2037	2032
Eswatini	2033	2033	2030
Ethiopia	2041	2043	2035
Fiji	2033	2033	2030
Finland	2036	2037	2032
France	2036	2038	2031
Gabon	2040	2042	2034
Gambia, The	2038	2039	2034
Georgia	2035	2035	2031
Germany	2034	2035	2030
Ghana	2035	2035	2032
Greece	2036	2037	2032
Grenada	2039	2040	2033
Guam			
Guatemala	2038	2039	2032
Guinea	2045	2048	2037
Guinea-Bissau	2038	2039	2034
Guyana	2035	2035	2031
Haiti	2042	2044	2035
Honduras	2038	2039	2032
Hungary	2038	2040	2033
Iceland	2038	2040	2033
India	2035	2035	2031
Indonesia	2042	2045	2035
Iran, Islamic Rep.	2038	2039	2032
Iraq	2038	2039	2032
Ireland	2036	2037	2032
Israel	2036	2037	2032
Italy	2034	2035	2030
Jamaica	2038	2039	2032
Japan	2038	2040	2033
Jordan	2041	2044	2034
Kazakhstan	2038	2040	2033

	Year of Introduction for the Pharma MNC Global Rollout Scenario		
	Base Case Timeline Scenario	Conservative Timeline Scenario	Optimistic Timeline Scenario
Kenya	2035	2035	2032
Kiribati	2033	2033	2030
Korea, Dem. People's Rep.	2041	2042	2035
Korea, Rep.	2037	2039	2032
Kuwait	2036	2037	2032
Kyrgyz Republic	2038	2038	2033
Lao PDR	2040	2042	2034
Latvia	2036	2037	2032
Lebanon	2041	2044	2034
Lesotho	2038	2039	2034
Liberia	2038	2039	2034
Libya	2038	2039	2032
Lithuania	2036	2037	2032
Luxembourg	2036	2037	2032
Madagascar	2038	2039	2034
Malawi	2035	2035	2032
Malaysia	2039	2040	2033
Maldives	2039	2040	2033
Mali	2038	2039	2034
Malta	2039	2041	2033
Mauritania	2038	2039	2034
Mauritius	2035	2035	2031
Mexico	2039	2041	2033
Micronesia, Fed. Sts.	2036	2037	2032
Moldova	2036	2037	2032
Mongolia	2037	2038	2032
Montenegro	2043	2046	2035
Morocco	2035	2035	2031
Mozambique	2038	2039	2034
Myanmar	2039	2040	2034
Namibia	2036	2037	2032
Nepal	2039	2040	2034
Netherlands	2038	2040	2033
New Zealand	2034	2035	2030
Nicaragua	2035	2035	2031
Niger	2038	2039	2034
Nigeria	2040	2042	2034
North Macedonia	2040	2042	2034
Norway	2036	2037	2032
Oman	2038	2040	2033

	Year of Introduction for the Pharma MNC Global Rollout Scenario		
	Base Case Timeline Scenario	Conservative Timeline Scenario	Optimistic Timeline Scenario
Pakistan	2040	2041	2034
Panama	2038	2039	2032
Papua New Guinea	2039	2042	2032
Paraguay	2038	2039	2032
Peru	2038	2039	2032
Philippines	2044	2047	2036
Poland	2038	2040	2033
Portugal	2038	2040	2033
Qatar	2036	2037	2032
Romania	2042	2045	2035
Russian Federation	2039	2041	2033
Rwanda	2035	2035	2032
Samoa	2043	2046	2035
São Tomé and Principe	2033	2033	2030
Saudi Arabia	2036	2037	2032
Senegal	2035	2035	2032
Serbia	2038	2040	2033
Seychelles	2035	2035	2031
Sierra Leone	2035	2035	2032
Singapore	2038	2040	2033
Slovak Republic / Slovakia	2038	2040	2033
Slovenia	2038	2040	2033
Solomon Islands	2035	2036	2031
Somalia	2045	2048	2037
South Africa	2035	2036	2031
South Sudan	2045	2048	2037
Spain	2036	2038	2031
Sri Lanka	2040	2042	2034
St. Lucia	2038	2039	2032
St. Vincent and the Grenadines	2039	2040	2033
Sudan	2037	2037	2033
Suriname	2042	2044	2035
Sweden	2038	2040	2033
Switzerland	2038	2040	2033
Syrian Arab Republic	2047	2050	2038
Tajikistan	2039	2040	2034
Tanzania	2038	2039	2034
Thailand	2041	2043	2034
Timor-Leste	2041	2043	2034
Togo	2038	2039	2034

	Year of Introduction for the Pharma MNC Global Rollout Scenario		
	Base Case Timeline Scenario	Conservative Timeline Scenario	Optimistic Timeline Scenario
Tonga	2035	2036	2031
Trinidad and Tobago	2037	2038	2032
Tunisia	2040	2042	2034
Turkey	2038	2040	2033
Turkmenistan	2037	2038	2032
Uganda	2035	2035	2032
Ukraine	2043	2046	2035
United Arab Emirates	2035	2035	2031
United Kingdom	2033	2034	2029
United States	2033	2034	2029
Uruguay	2038	2040	2033
Uzbekistan	2035	2035	2031
Vanuatu	2037	2038	2032
Venezuela, RB	2044	2048	2036
Vietnam	2043	2046	2035
Yemen, Rep.	2040	2041	2034
Zambia	2038	2039	2034
Zimbabwe	2035	2035	2032

Timing of Introduction Analysis Results

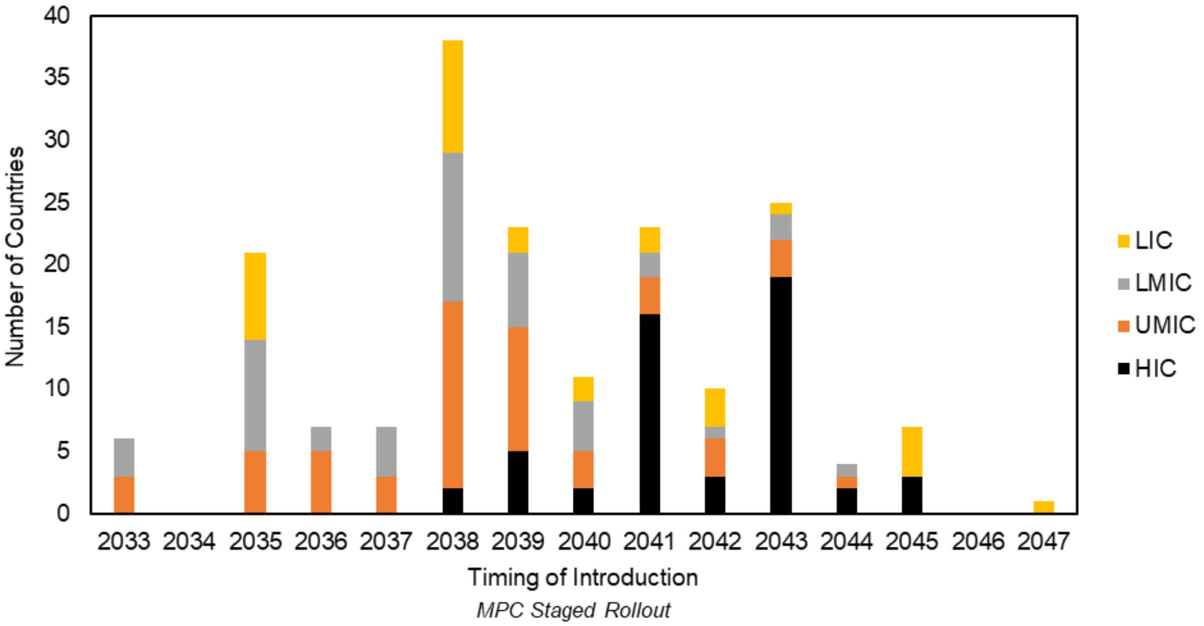

Supplementary Figure 1. Overview of timing of introduction in the public market (national immunization program; NIP), segmented by country-income level group for the **Pharma MNC** Staged Rollout scenario (base case timeline).

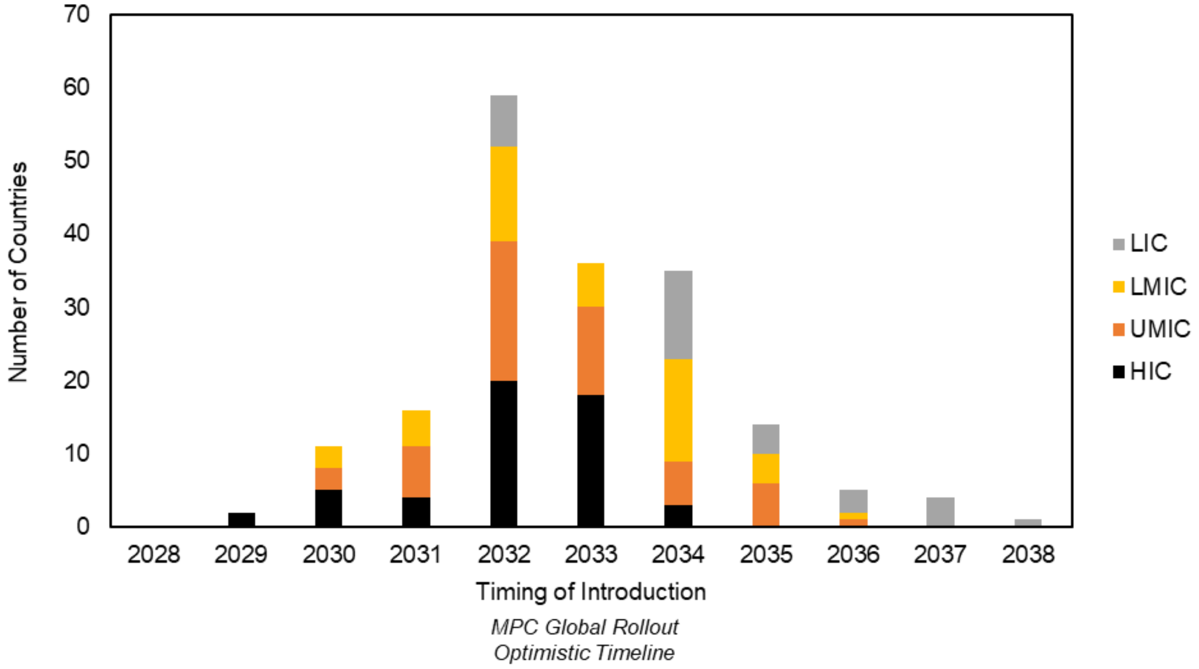

Supplementary Figure 2. Overview of timing of introduction in the public market (national immunization program; NIP), segmented by country-income level group for the **Pharma MNC** Global Rollout scenario under the Optimistic Timeline scenario.

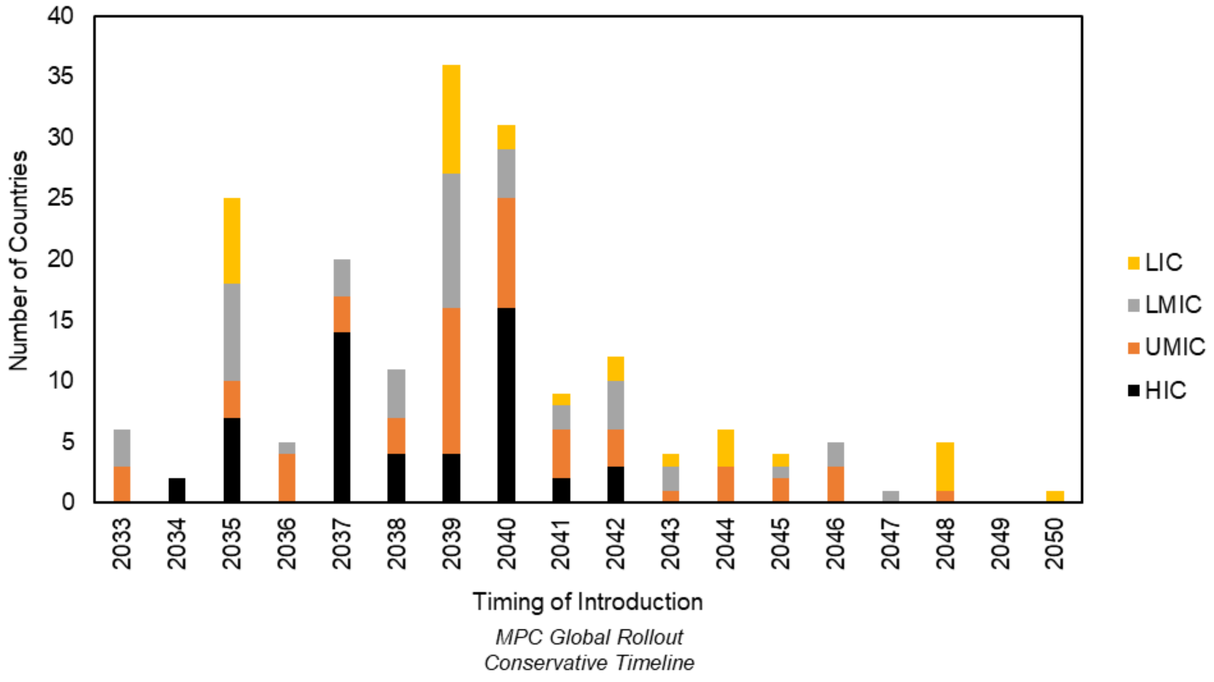

Supplementary Figure 3. Overview of timing of introduction in the public market (national immunization program; NIP), segmented by country-income level group for the **Pharma MNC** Global Rollout scenario under the Conservative Timeline scenario.

Demand and Financial Forecasts

Supplementary Table 7. Overview of year 12 vaccine demand and financial forecasts for the **Pharma MNC** Global Rollout vs. **Pharma MNC (no HIC markets) vs. Pharma MNC** Staged Rollout scenarios (infant immunization program, Base Case timeline).

	**Pharma MNC Global Rollout**	**Pharma MNC (no HIC markets)**	**Pharma MNC Staged Rollout**
**Year 12 Annual Demand (number of doses)**	298M	264M	286M
**Year 12 Annual Revenue**	$3.9B	$2.0B	$3.1B
**Year 12 Annual Profit**	$2.9B	$1.1B	$2.3
**Year 12 Gross Profit Margin**	75%	57%	71%
**Year 12 NPV**	$2.5B	$0.62B	$1.3B

Supplementary Table 8. Overview of year 12 vaccine demand and financial forecasts for the **Pharma MNC** Global Rollout scenario with Base Case, Optimistic and Conservative timelines (infant immunization program).

	**Base Case Timeline**	**Optimistic Timeline**	**Conservative Timeline**
**Year 12 Annual Demand (number of doses)**	298M	370M	189M
**Year 12 Annual Revenue**	$3.9	$4.4B	$2.5
**Year 12 Annual Profit**	$2.9	$3.2B	$1.9B
**Year 12 Gross Profit Margin**	75%	73%	76%
**Year 12 NPV**	$2.5B	$6.7B	$1.5B
